# Community-based exercises improve health status in pre-frail older adults: A systematic review with meta-analysis

**DOI:** 10.1186/s12877-024-05150-7

**Published:** 2024-07-10

**Authors:** Huijun Lim, Nur Dalilah Binte Jani, Wai Teng Pang, Edwin Choon Wyn Lim

**Affiliations:** 1 New Hope Community Services, Yishun, Singapore; 2 SingHealth Polyclinic, Punggol, Singapore; 3 HCA Hospice Limited, Serangoon, Singapore; 4 Health and Social Sciences Cluster, Singapore Institute of Technology, Dover, Singapore; 5 Active Global Home and Community Care, 51 Goldhill Plaza, #12-11, Novena, 308900 Singapore

**Keywords:** Exercise, Physical activity, Physical function, Cognition, Quality of life, Pre-frailty, Frailty

## Abstract

**Background:**

Pre-frailty is associated with increased healthcare utilization. Over the past decade, public health interventions such as community-based exercises to target pre-frailty have been increasingly studied. However, the effects of community-based exercises on clinical outcome measures amongst community-dwelling older adults with pre-frailty remain unclear. This review aims to better understand the effects of community-based exercise on physical function, cognition, quality of life and frailty status in community-dwelling pre-frail older adults. A secondary objective was to investigate the optimal exercise parameters on clinical outcomes.

**Methods:**

Searches of MEDLINE, CINAHL, Google Scholar and Web of Science databases were conducted. Articles were included if they were randomized controlled trials (RCTs), and excluded if the participants consist of less than 50% pre-frail community-dwelling older adults. Meta-analyses (where possible) with either a fixed- or random- effect(s) model, standardized mean difference (SMD), odds ratio (OR) and tests of heterogeneity were performed. Multivariable meta-regression was performed to identify predictors of statistically significant outcome measures. The risk of bias was assessed using the modified Cochrane Risk-of-Bias tool.

**Results:**

Twenty-two RCTs with 900 participants in the experimental group and 1015 participants in the control group were included. When compared to minimal intervention, community-based exercises significantly improved lower limb strength (10 RCTs, 384 participants in the experimental group and 482 participants in the control group) with SMD 0.67 (95% CI 0.29 to 1.04), and lower limb function (5 RCTs, 120 participants in the experimental group and 219 participants in the control group) with SMD 0.27 (95% CI 0.03 to 0.51). Those who have received community-based exercises were more likely to reverse from pre-frailty to healthy state (OR = 2.74, 95% CI 1.36 to 5.51) (6 RCTs, 263 participants in the experimental group and 281 participants in the control group). The frequency of exercise sessions was a significant predictor of the effect size for gait speed (*P*<0.05).

**Conclusions:**

Community-based exercise intervention is superior to minimal intervention for improving health status in pre-frail older adults. This has implications on the implementation of community-based exercise intervention by healthcare providers and policymakers.

**Other:**

Nil funding for this review. PROSPERO registration number CRD42022348556.

**Supplementary Information:**

The online version contains supplementary material available at 10.1186/s12877-024-05150-7.

## Introduction

Pre-frailty is prevalent amongst older adults [1], and it reportedly poses a socioeconomic burden such as healthcare costs on the society [2, 3]. It is an early and reversible risk-state of health before frailty which can lead to negative healthcare outcomes such as falls, cognitive decline, hospitalization or even death [4–6]. Thus far, the interventions for addressing pre-fraily included physical activity, nutrition, and physical activity combined with nutrition [7].

With the ongoing rise in life expectancy worldwide [8], there is increasing public health focus, at least in Singapore, on healthy aging through physical activity such as community-based exercises to maintain independence among older adults [9]. In recent years, community-based exercises amongst older adults with pre-frailty have been increasingly studied [9–11]. Community-based exercises also provide an opportunity to stimulate social engagement amongst older adults [12]. The availability of community-based exercises has brought about convenience to the older adults due to increased accessibility [12]. To date, the average adherence rates of community-based exercise for older adults has been estimated to be approximately 70% by a previous study [13]. However, the evidence on the effectiveness of community-based exercises on clinical measures in pre-frailty appears mixed or unclear. For example, significant changes in grip strength have been reported in two trials [14, 15], but not in two other trials[16, 17]. Secondly, there are systematic reviews which investigated the effects of exercise intervention on physical measures in pre-frailty. Two of them were descriptive in nature [7, 18], whilst another review did not manage to investigate physical outcome measures such as strength, balance and walking speed [19].

Therefore, we aimed to review randomized controlled trials comparing the effects of community-based exercise (intervention) with minimal intervention on physical function, cognition and quality of life (outcome) in community-dwelling pre-frail older adults (participants). A secondary objective was to investigate the influence of parameters such as frequency of sessions per week, and total number of sessions on the effect size of statistically significant outcome measures.

## Methods

The protocol of this study was published at PROSPERO (http://www.crd.york.ac.uk/PROSPERO/; registration number CRD42022348556). This review was also completed in accordance to the Preferred Reporting Items for Systematic Reviews and Meta-Analyses (PRISMA) guidelines [20].

### Search strategy

We searched MEDLINE (1966-present), CINAHL (1966-present), Google Scholar and Web of Science, for literature on the effects of community-based exercise on physical function, cognition, frailty status and quality of life in community-dwelling older adults with pre-frailty (Supplementary Fig. 1). The last search was run on Sep 16, 2023. The following search terms were used to search the databases: group exercise; physical activity; community*; pre-frail*; randomized controlled trial. These steps were then repeated for the other databases. The reviewers followed a selection process, defined prior to the beginning of the review, which included a checklist for inclusion criteria. Articles were eligible for inclusion if they were randomized controlled human trials, included community-dwelling pre-frail older adults aged 60 years and above, assigned the experimental group to receive treatment which includes at least exercise, assigned the comparison group to receive other forms of intervention other than exercise, and lastly, used outcome measures that included physical function, cognition, quality of life and/or frailty status. We also included trials with at least 50% or more older adults with pre-frailty. Participants were considered pre-frail if pre-frailty has been mentioned explicitly by the authors and/or determined via the use of screening tools such as Fried’s frailty criteria [21], FRAIL questionnaire [22], and Cardiovascular Health Study criteria [23]. Pre-frailty is herein defined as having met 1 or 2 criteria with reference to an established set of indicators in the aforementioned screening tools such as unintended weight loss, self-reported exhaustion, poor handgrip strength, slow walking speed, and low physical activity [24]. Articles were excluded if the participants consist of less than 50% pre-frail community-dwelling older adults,did not include outcome measures such as physical function, cognition, frailty status and quality of life as outcome measures, and/or the participants were hospitalised or institutionalized. Eligibility assessment for included studies was determined by 2 reviewers (H.J.L. and E.C.W.L). Disagreements between reviewers were resolved by consensus with another 2 independent reviewers (W.T.P. and N.D.J.).

### Data extraction and quality of trials assessment

The methodological quality of the trials was assessed using the 11-item PEDro scale [25]. We assessed the methodological quality of the studies by evaluating the domains of population, treatment allocation, blinding, prognostic comparability, and analysis. Using a standardized extraction form, information on the characteristics of trial participants (age and gender), details of intervention (type of exercise, number of sessions per week, duration of session in minutes, and time span of exercise program in weeks), and outcome measures (pre- and post-intervention) were extracted from each included trial. The assessment methodological quality and extraction of data were performed and verified between 2 reviewers (H.J.L. and E.C.W.L). Differences between reviewers were resolved by agreement with another 2 independent reviewers (W.T.P. and N.D.J.).

The outcome measures included herein in our review were hand grip strength [22, 26], functional lower limb strength measures such as timed 5-times sit-to-stand [27], 30 s chair rise test [28], and Short Physical Performance Battery (SPPB) chair rise score [29], functional balance measures such as timed one-legged stance [30] and SPPB balance score [29], gait speed such as 4- to 6-m walk test [31–33] and SPPB gait score [29], Timed Up And Go test [34], SPPB overall score [29], functional exercise capacity such as 2-min walk test [35] and 6-min walk test [36], cognitive measures such as Mini-Mental State Examination [37], Montreal Cognitive Assessment [38], Frontal Assessment Battery [39], and Repeatable Battery for the Assessment of Neuropsychological Status [40], quality of life such as EuroQoL-5D [41], 36-Item Short Form Health Survey [42], Quality of life visual analogue scale [43] and Life Satisfaction score [44], and the number of participants with reversal of pre-frailty status.

The risk of bias was assessed with the use of revised Cochrane risk-of-bias tool [45]. It evaluates risk of bias in 5 distinct domains, that is, the randomization process, deviations from intended interventions, missing outcome data, measurement of the outcome, and selection of the reported result [45]. If the outcome measures were reported for more than one side and/or multiple time points, then the pre- and post-intervention outcome measures which gave the worst mean difference (MD) were extracted [46]. The outcome scores were approximated with the use of available median value, range, interquartile range, and standard error, whenever they were not presented in mean and/or standard deviation [47–49]. 

### Quantitative data synthesis and analysis

Reliability analyses of inter-rater agreement were performed with IBM SPSS Statistics for Windows, Version 21.0 (IBM Corp, Armonk, NY). Inter-rater reliability was reported for the total quality score with Kappa statistics,[50] and was interpreted as poor (< 0.00), slight (0.00–0.20), fair (0.21–0.40), moderate (0.41–0.60), substantial (0.61–0.80), or almost perfect (0.81–1.0). Where appropriate and possible, the results were pooled with formal meta-analytical techniques using RevMan 5.4.1 (The Nordic Cochrane Centre, The Cochrane Collaboration, Copenhagen, Denmark). To account for differing outcome scales used among studies, we calculated standardized mean differences (SMDs) for the outcome scores, their 95% confidence intervals (CIs), and performed tests of heterogeneity (χ^2^). The *I*^*2*^ statistic was used to measure the extent of between-trial heterogeneity. Fixed-effect or random-effects models were used as appropriate and were based on our interpretation of commonality of effect size[51]. For example, data were pooled using a random-effects model, if trials differed in ways that might have plausibly impacted on the pooled outcome [51].

For continuous data, the differences in pre- and post-intervention pain score were calculated such that positive values indicated that the results favored community-based exercises, whilst negative values indicated that the results favored minimal intervention. We used odds ratios and 95% CIs to calculate the intervention effects for dichotomous data such as frailty status, and the number needed to treat [52]. Post-hoc sensitivity analyses were performed in the presence of apparent outliers.

Multivariable regression analyses were repeated to investigate if the commonly reported temporal parameters, that is, frequency, time span and duration predict the effect size for outcome measures which yielded statistically significant pooled result, and have at least 10 available trials [53]. The assumptions of this regression model were verified by examining the normal predicted probability plot, scatterplot of predicted values versus residuals, and variance inflation factor. For all analyses, significance was set at *P* < 0.05. To evaluate the risk of publication bias (due to non-publication of small trials with negative results), we plotted SMD versus SE and visually assessed the symmetry of this ‘funnel’ plot.

### Quality of evidence assessment

The Grading of Recommendations Assessment, Development and Evaluation (GRADE) system was used to determine the overall quality of evidence for variables used in meta-analyses. GRADE considers five criteria (risk of bias, publication bias, imprecision, inconsistency and indirectness) to rate the quality of evidence as high, moderate, low or very low. In the GRADE approach, randomized controlled trials start as high-quality evidence and observational studies as low-quality evidence supporting estimates of intervention effects. The quality of evidence was rated up or down by two independent reviewers (H.L and E.C.W.L) for certain factors and the lowest quality of evidence among the criteria is considered the overall quality of evidence.

## Results

### Study selection

**Fig. 1:**
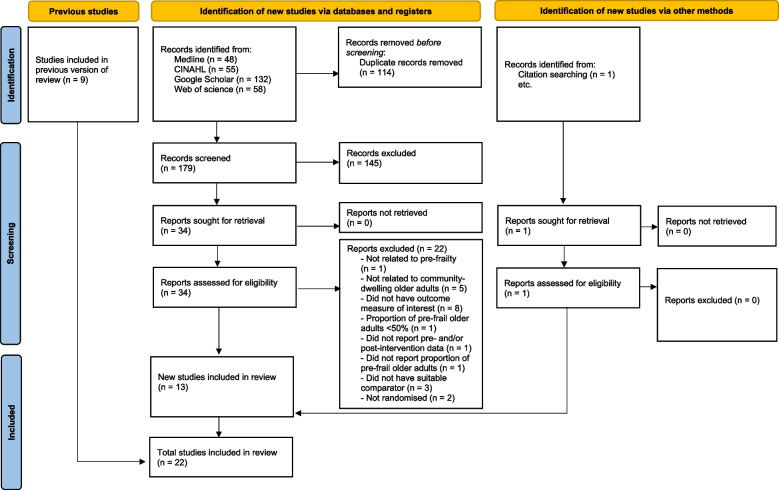
Selection process for studies included in analysis

A total of 293 articles emerged from the inceptive electronic database search; of these, 34 were assessed for eligibility, and 22 eligible papers were included in this review. Fig. 1 displays the flow of papers through review. The basis for exclusion of articles after retrieval and assessment of eligibility included non-relevance to pre-frailty [54], non-relevance to community-dwelling older adults [55–59], lack of outcome measures of interest [23, 60–66], failure to meet desired representation of participants [67], lack of reporting on the proportion of pre-frail community-dwelling adults [68], lack of reporting on the pre- and/or post-intervention data [69], lack of suitable comparator [70–72], and non-randomized controlled trials [9, 16].

### Methodological quality

There was substantial concurrence between the 2 reviewers (κ = 0.802, *P* < 0.001). Individual item agreement percentages ranged from 72.7% to 100%. The methodological quality assessment using the PEDro scale yielded a mean score of 6.45 (range = 3–9) out of a possible 10 points (Table 1). Criteria commonly not met were concealment of allocation, blinding of treating therapists or patients, and intention-to-treat analysis.

**Table 1:** Details of the included randomized controlled trials

Authors (Year), Study design	Intervention (n)	Age (years)	Gender (M, F)	Pre-frail, n (%)	Brief details of intervention	Pre-intervention, means (SD)	Post-intervention, means (SD)	Effect Measure—Mean difference between groups or Odds ratio (95% CI)	PEDro score (/10)	Cochrane's Risk of Bias 2
Barrachina-Igual [ 21] , RCT						(baseline)	(post-intervention)	Grip strength 1.75 (-2.45, 5.95) SPPB 0.51 (-0.61, 1.63) Odds ratio—pre-frailty reversal 3.64 (0.82, 16.10)	6	Some concerns
	Intervention (*n* = 23)	74.8 (5.78)	M 7, F 16	14 (61.0)	Multi-component training program (i) 10 min warm up which included brisk walk and joint mobility exercises, (ii) Progressive high-intensity resistance training using a circuit (iii) Self-massage for myofascial release (iv) Cool down which included static stretches 65 min each session, 2x/week over 12 weeks	Maximum hand grip strength, kg				
						25.5 (6.42)	26.6 (7.69)			
						SPPB				
						10.17 (1.74)	10.78 (1.24)			
						Number of pre-frail who transitioned to robust, n				
						9				
	Control (*n* = 20)	75.3 (8.2)	M 5, F 15	13 (65.0)	Continue with routine daily activities	Maximum hand grip strength, kg				
						26.5 (6.68)	25.85 (7.08)			
						SPPB				
						9.65 (2.23)	9.75 (1.97)			
						Number of pre-frail who transitioned to robust, n				
						3				
Bray [26], Quasi-experimental						(baseline)	(post-intervention)		3	High
	Exercise (*n* = 8)	72.9 (4.8)	M 0, F 8	5 (62.5)	Multi-component exercise (i) Aerobic warm up which included treadmill, stationary bike, elliptical and marching in place, (ii) Resistance training with the use of dumbbells which included squat, deadlift, bench press and leg press; with weight increased when participant performed 3 sets with upper limit of repetition range, (iii) Balance exercise, (iv) Flexibility cool-down, 45–60 min, 3x/wk over 12 weeks with > 80% adherence to participation in exercise	Grip strength, kg		Grip strength 4.04 (-0.61, 8.69) 5 times sit-to-stand time -3.06 (0.53, 5.59) Gait speed 0.27 (0.07, 0.47) Odds ratio—pre-frailty reversal 2.78 [0.37, 21.03]		
						24.9 (4.72)	28.8 (6.41)			
						5 times sit-to-stand time, sec				
						15.2 (2.93)	10.2 (2.35)			
						Gait speed, m/sec				
						1.1 (0.28)	1.34 (0.22)			
						Number of pre-frail who transitioned to robust, n				
						5				
	Control (*n* = 8)	72.4 (5.4)	M 0, F 8	4 (50.0)	Multi-component exercise (As above) with < 80% adherence to participation in exercise + maintained their normal routine	Grip strength, kg				
						24.2 (3.43)	24.0 (3.46)			
						5 times sit-to-stand time, sec				
						11.4 (2.83)	9.43 (1.07)			
						Gait speed, m/sec				
						1.24 (0.16)	1.21 (0.1)			
						Number of pre-frail who transitioned to robust, n				
						3				
Chan [73], RCT						(baseline)	(after 12 months)	Left one leg stand time 0.26 (-3.06, 3.58) MMSE -0.21 (-1.13, 0.71)	6	Some concerns
	Exercise and nutritional program (*n* = 55)	70.9 (3.7)	M 22, F 33	46 (84.0)	(i) 15 min warm up which included10 min brisk walk, gentle stretches, (ii) 20–30 min resistance training with the use of rubber band and bottled water, (iii) 10 min postural control activities and balance training which included tandem gaits, one leg standing, step up and down stairs, toe walking and heel walking, (iv) 5 min cool-down, 3x/wk over 3 months	Left one leg stand time, sec*				
						Mean change (SD) 3.69 (9.15)				
						MMSE*				
						Mean change (SD) -0.15 (2.53)				
	Education booklet (*n* = 62)	71.9 (3.7)	M 26, F 36	56 (90.0)	Education booklet on frailty, healthy diets, exercise protocols and self-coping strategies	Left one leg stand time, sec*				
						Mean change (SD) 3.43 (9.15)				
						MMSE*				
						Mean change (SD) 0.06 (2.52)				
Chan and Yu [74], RCT,						(baseline)	(after 12 weeks)	8.30 (-0.4, 17.0)	7	High
	Aerobic exercise (*n* = 61)	77.2 (7.3)	M 9, F 52	42 (68.9)	(i) 10 min warm-up, (ii) 30 min of moderate-intensity low-impact stepping exercise, (iii) 10-min cool-down, 3x/wk over 12 weeks	Two-minute walk test, metres*				
						83.1 (23.9)	92.8 (25.7)			
	Health education (*n* = 63)	78.1 (7.4)	M 8, F 55	40 (63.5)	Health-education program bi-weekly which included health talks on topics such as medication safety, falls prevention and home safety, pain management, dietary management, dementia and cataracts, 60 min each session over 12 weeks	Two-minute walk test, metres*				
						81.7 (24.8)	83.1 (24.3)			
Chen [14], RCT						(baseline)	(after 8 weeks)	Grip strength 4.84 [3.34, 6.34] Walking time -1.27 [-1.70, -0.84]	7	Some concerns
	Elastic band (*n* = 21)	77.0 (5.19)	M 12, F 21	43 (100.0)	Elastic band exercise (warm-up, 8 exercise movements, and relaxed activities), 45–60 min per session, 3x/wk over 8 weeks	Grip strength, kg				
						25.9 (3.06)	30.8 (4.11)			
						Walking time, sec				
						5.59 (0.91)	4.32 (0.57)			
	Control (*n* = 22)	75.3 (5.98)	M 11, F 22		Maintained normal daily activity and did not receive any special intervention, continued with irregular exercise (less than 2 days a week) or remained sedentary	Grip strength, kg				
						26.8 (2.44)	26.8 (2.26)			
						Walking time, sec				
						5.98 (0.95)	5.98 (0.97)			
Chittrakul [75], RCT,						(baseline)	(after 12 weeks)	13.10 (8.92, 17.3)	8	Some concerns
	Multi-System Physical Exercise (*n* = 36)	69.1 (3.55)	Not reported‡	36 (100.0)	(i) Proprioceptive training which included seated ankle ball and single leg stance, (ii) Muscle strength training which included sit-to-stand, knee raise standing and squats, (iii) Reaction exercise training with auditory cues which included step up, step forward, step backward and step to the sides, and (iv) Postural balance training which included heel-to-toe standing, side leg raise, heel raises, heel walking, toe walking, and 8-shaped walking, 60 min, 3x/wk over 12 weeks	HRQOL				
						67.4 (10.82)	93.9 (9.0)			
	Control (*n* = 36)	68.9 (3.86)		36 (100.0)	Received flexibility exercise training, 3x/wk over 12 weeks	HRQOL				
						77.0 (8.66)	90.4 (1.68)			
Dun [10], RCT						(pre-test)	(after 12 sessions)	30 s arm curl test 8 (6.0, 10.0) 30 s chair stand test 7.0 (5.0, 9.0) 2.4 m up-and-go -1.8 (-2.4, -1.2) 6-min walk distance 86.0 (55.0, 117.0) Odds ratio—pre-frailty reversal 126.67 [12.1, 1326.39]	7	Low
	X-CircuiT (*n* = 24)	72.0 (6.0)	M 9, F 15	48 (100.0)	(i) 4.5-min warm-up which included stationary light march, neck stretches shoulder shrugs etc., (ii) 6.5-min aerobic training which included lateral steps, lateral cross, knee ups, forward lunges and front steps, (iii) 6-min acupoint patting, (iv) 15-min elastic resistance training which included shoulder abduction, bicep curl, half-squat, hip abduction etc., (v) 14-min flexibility training which included shoulder flexion, shoulder abduction, trunk stretch, gluteus stretch, hamstring stretch etc., 3x/wk over 3 months	30 s arm curl test, reps				
						Mean change (95% CI) 8.0 (7.0, 9.0)				
						30 s chair stand test, reps				
						Mean change (95% CI) 5.0 (3.0, 7.0)				
						2.4 m up-and-go, seconds				
						Mean change (95% CI) -1.1 (-1.4, -0.8)				
						6-min walk distance, meters				
						Mean change (95% CI) 68.0 (50.0, 86.0)				
						Number of pre-frail who transitioned to robust, n				
						19 (out of 22)				
	Control (*n* = 24)	73.0 (6.0)	M 8, F 16		One-off advice on physical activity according to current evidence (150 min or more per week of moderate to vigorous physical activity) without supervised exercise	30 s arm curl test, reps				
						Mean change (95% CI) -1.0 (-3.0, 1.0)				
						30 s chair stand test, reps				
						Mean change (95% CI) -1.0 (-3.0, 0.0)				
						2.4 m up-and-go, seconds				
						Mean change (95% CI) 0.7 (0.1, 1.3)				
						6-min walk distance, meters				
						Mean change (SD) -16.0 (-50.0, 18.0)				
						Number of pre-frail who transitioned to robust, n				
						1 (out of 21)				
Ge [ 76], RCT						(baseline)	(after 8 weeks)	30-s chair rise test 4.91 [3.64, 6.18] Walking speed 0.82 [0.34, 1.30]	6	Some concerns
	Tai Chi (*n* = 32)	70.2 (5.4)	M 21, F 11	65 (100.0)	(i) Warm-up for 10 min which focused on muscle stretching and joint activities, (ii) First eight of 24-form Yang-style Tai Chi which are organised in a sequential format that is easy for learners to follow, (iii) Cool-down for 10 min which included meditation, imagining learned Tai Chi moves and deep breathing exercises while standing, 3x/wk over 8 weeks	30-s chair rise test, reps*				
						12.2 (2.57)	17.3 (2.0)			
						Walking speed, m/s*				
						4.73 (0.68)	3.94 (0.59)			
	Control (*n* = 33)	72.9 (6.6)	M 16, F 17		Maintained normal daily activities and did not receive any special intervention	30-s chair rise test, reps*				
						11.2 (2.8)	11.4 (2.94)			
						Walking speed, m/s*				
						5.14 (1.29)	5.17 (1.22)			
Huguet [77], RCT						(baseline)	(post-intervention)	Five times sit-to-stand -2.00 (-0.28, -3.72) TUG -1.60 (-3.07, -0.13) Odds ratio—pre-frailty reversal 14.3 [1.82, 112.61]	5	High
	Intervention (*n* = 100)	84.5 (3.4)	M 32, F 68	200 (100.0)	(i) Assessment of polypharmacy (ii) Group session on nutrition (iii) Physical exercise program; aerobic exercise (walking 30–60 min a day for 3x/wk), strengthening, and balance and coordinations exercises, fortnightly for 9 sessions over 6 months, (iv) Review of personal and environmental conditions and social support	Five times sit-to-stand, sec				
						19.6 (6.8)	17.0 (6.0)			
						TUG				
						13.4 (4.3)	12.4 (4.2)			
						Number of pre-frail who transitioned to robust, n				
						12 (out of 85)				
	Control (*n* = 100)	84.5 (3.7)	M 39, F 61		Standard public health centre treatmemt from family physicians, nurses and social workers	Five times sit-to-stand, sec				
						18.3 (5.2)	17.7 (4.8)			
						TUG				
						13.4 (5.1)	14.0 (5.9)			
						Number of pre-frail who transitioned to robust, n				
						1 (out of 88)				
Jiayuan [11], RCT						(baseline)	(after 6 months)	30 s chair stand test 1.2 (0.2, 2.2) SPPB 0.5 (-0.06, 1.06) TUG -1.3 (-1.64, -0.96) MMSE 0.0 (-1.05, 1.05) Odds ratio—pre-frailty reversal 6.0 [1.17, 30.72]	9	Some concerns
	Mindfulness (*n* = 30)	70.8 (4.2)	M 13, F 17	18 (60.0)	Booklet about mindfulness skills, 10-min review on problem solving, 45-min exercises on mindfulness, 5-min summary, 2x/wk over 6 months	30 s chair stand test, reps				
						16.6 (2.1)	16.7 (1.9)			
						SPPB				
						9.1 (1.1)	9.4 (1.0)			
						TUG				
						10.7 (0.6)	10.5 (0.7)			
						MMSE				
						23.9 (2.6)	25.1 (2.4)			
						Number of pre-frail who transitioned to robust, n				
						9				
	Mindfulness-based Tai Chi Chuan (*n* = 30)	71.3 (5.0)	M 13, F 16	17 (56.7)	Booklet about mindfulness skills (as above), Tai Chi Chuan (i) 10-min warm-up which included muscle stretching and joint movement, (ii) 45-min physical exercise, (iii) 5-min cool-down which included deep breathing and relaxation, 2x/wk over 6 months	30 s chair stand test, reps				
						16.1 (2.0)	17.4 (1.9)			
						SPPB				
						9.2 (1.2)	10.0 (1.1)			
						TUG				
						10.6 (0.8)	9.1 (0.5)			
						MMSE				
						24.5 (1.6)	25.7 (1.5)			
						Number of pre-frail who transitioned to robust, n				
						2				
Kwon [78], RCT						(baseline)	(after 3 months)		6	Some concerns
	Exercise training and nutrition (*n* = 26)	76.5 (3.8)	M 0, F 26	79 (100.0)	Exercise training (i) 10–15 min of warm-up and stretches, (ii) 20–45 min of strengthening with the use of Thera bands, dumbbells and balls, and balance exercises, and (iii) 5–10 min of cool-down), over 12 weeks Nutritional intervention program (cooking practice) to acquire an eating habit that helps to strengthen muscles	Handgrip strength, kg*		Exercise training vs Control Handgrip strength 0.80 [-1.01, 2.61] Stork stand time with eyes open -1.60 [-9.56, 6.36] Usual walking speed -0.04 [-0.31, 0.23]		
						Mean change (SD) 1.2 (4.0)				
						Stork stand time with eyes open, secs*				
						Mean change (SD) 2.9 (18.6)				
						Usual walking speed, m/s*				
						Mean change (SD) 0.17 (0.34)				
	Exercise training (*n* = 25)	77.0 (4.2)	M 0, F 25		Exercise training (as above)	Handgrip strength, kg*				
						Mean change (SD) 2.3 (3.1)				
						Stork stand time with eyes open, secs*				
						Mean change (SD) -2.0 (16.9)				
						Usual walking speed, m/s*				
						Mean change (SD) 0.09 (0.59)				
	Control (*n* = 28)	76.9 (3.9)	M 0, F 28		General health education session which provided information on physical training for falls prevention, urinary incontinence and dietary guideline for healthy aging, once/month over 3 sessions	Handgrip strength, kg*				
						Mean change (SD) 0.4 (2.6)				
						Stork stand time with eyes open, secs*				
						Mean change (SD) -0.4 (11.9)				
						Usual walking speed, m/s*				
						Mean change (SD) 0.13 (0.38)				
Lustosa [79], RCT						(baseline)	(post-intervention)	Gait speed 0.46 [-0.08, 1.00] TUG 0.04 [-1.24, 1.32]	7	Some concerns
	Experimental (*n* = 32)	72.0 (4.0)	M 0, F 32	48 (100.0)	Training program which targeted the lower limbs, (i) Open kinetic chain exercises with the use of ankle weights (0.5 to 3 kg), (ii) Closed kinetic chain exercises, that is, semi-squat, intensity of 75% of repetition maximum, 1 h, 3x/wk over 10 weeks	Gait speed, sec				
						4.85 (0.7)	4.36 (0.7)			
						TUG, sec				
						11.1 (2.3)	10.4 (1.9)			
	Control (*n* = 16)	72.0 (3.5)	M 0, F 16		Remained with the same activities of normal life, without doing any training	Gait speed, sec				
						4.9 (1.1)	4.87 (0.8)			
						TUG, sec				
						10.8 (2.4)	10.1 (1.7)			
Meng [80], RCT						(baseline)	(after 3 months)	Grip strength -0.95 (-2.81, 0.91) Timed chair stand 0.54 (-0.01, 1.09) Walking speed 0.00 (-0.07, 0.07) TUG -0.36 (-0.68, -0.03) Single leg stance 0.37 (-1.03, 1.77) 6-min walk distance 10.2 (-13.2, 33.6)	6	Some concerns
	Supervised exercise (*n* = 74)	76.5 (6.47)	M 34, F 40	59 (79.7)	(i) 10-min warm-up and stretching activities which included light calisthenics and stretches for the major muscle groups, (ii) 30-min aerobic exercise on a lower limb cycle ergometer with exercise intensity of 70% to 85% of predicted maximum heart rate, (iii) Resistance training which included elbow flexor strengthening with the use of dumbbells, hand grip strengthening and leg press), 1.5 h each session, 3x/wk over 3 months	Grip strength, kgw				
						24.1 (7.35)	25.1 (7.15)			
						Timed chair stand, sec				
						6.38 (3.01)	5.86 (2.71)			
						Walking speed, m/s				
						0.73 (0.23)	0.79 (0.23)			
						TUG				
						9.65 (5.28)	8.69 (4.26)			
						Single leg stance, sec				
						3.89 (3.47)	3.86 (4.68)			
						6-min walk distance, meters				
						398.5 (116.2)	405.6 (116.8)			
	Home-based exercise (*n* = 72)	76.7 (7.3)	M 30, F 42	54 (75.0)	15-min of home-based exercise instructions on calisthenics with handouts provided, (i) Gentle stretching exercises for all limbs and the trunk, and (ii) Resistance exercise for the upper and lower limbs such as dumbbell weight lifting, push-ups against wall, sit-to-stand, gentle semi-squatting, instructed to exercise at least 3 times per week at home	Grip strength, kgw				
						23.7 (7.82)	25.7 (9.75)			
						Timed chair stand, sec				
						6.19 (4.05)	6.2 (4.0)			
						Walking speed, m/s				
						0.71 (0.24)	0.78 (0.33)			
						TUG				
						10.2 (7.2)	10.6 (8.03)			
						Single leg stance, sec				
						4.02 (3.19)	4.35 (3.67)			
						6-min walk distance, meters				
						392.9 (116.8)	389.4 (129.3)			
Ng [81], RCT						(baseline)	(at 6-month follow-up)		8	Some concerns
	Combination (*n* = 49)	70.4 (4.74)	M 23, F 26	36 (73.5)	(i) Physical exercise which included resistance exercises integrated with functional tasks and balance training exercises involving functional strength, sensory input and added attentional demands, 8 to 15 repetition maximum or 60% to 80% of 10 repetition maximum, starting with less than 50% 1 repetition maximum involving 8–10 major muscles, 90 min, 2x/wk over 12 weeks (ii) Nutritional intervention which included a commercial formula taken daily for 24 weeks (iii) Cognitive training which included cognitive-enhancing activities designed to stimulate short-term memory, enhance attention, information-processing skills, reasoning and problem-solving abilities	Gait speed, sec*		Combination vs Control -0.16 [-0.75, 0.43] Physical training vs Control 0.40 [-0.19, 0.99]		
						Mean change (95% CI) -0.54 (-0.97, -0.1)				
	Physical training (*n* = 48)	70.3 (5.25)	M 21, F 27	29 (60.4)	Physical exercise (as above)	Gait speed, sec*				
						Mean change (95% CI) -1.1 (-1.53, -0.67)				
	Control (*n* = 50)	70.1 (5.02)	M 22, F 28	43 (86.0)	Access to standard care from health and aged care services; given equal volume of artificially sweetened, vanilla-flavoured liquid	Gait speed, sec*				
						Mean change (95% CI) -0.7 (-1.13, -0.27)				
Ng [23], RCT						(baseline)	(at 6-month follow-up)		8	High
	Combination (*n* = 49)	70.4 (4.74)	M 23, F 26	36 (73.5)	(i) Moderate intensity physical exercise as per American College of Sports Medicine Guidelines for older adults, 90 min, 2x/wk over 12 weeks (ii) Nutritional intervention (iii) Cognitive training (as per Ng 2015)	RBANS score		Combination vs Control 0.18 [0.03, 0.33] Physical training vs Control 0.14 [-0.01, 0.29]		
						Mean change (95% CI) 0.005 (-0.102, 0.112)				
	Physical training (*n* = 48)	70.3 (5.25)	M 23, F 25	29 (60.4)	Physical exercise (as above)	RBANS score*				
						Mean change (95% CI) -0.033 (-0.139, 0.072)				
	Control (*n* = 50)	70.1 (5.02)	M 21, F 29	43 (86.0)	Access to standard community-based social, recreational and day care rehabilitation services for older people; given placebo liquid capsules and tablet formulations	RBANS score*				
						Mean change (95% CI) -0.174 (-0.280, -0.067)				
Seino [82], RCT						(baseline)	(3 months post-intervention)	Hand grip strength -0.70 (-4.71, 3.31) Usual gait speed -0.01 (-0.12, 0.10) One-legged stance -4.50 (-15.29, 6.29) TUG -0.17 (-0.62, 0.28)	5	Some concerns
	Immedidate intervention (*n* = 38)	74.9 (5.3)	M 24, F 14	26 (68.4)	Multifactorial interventon (i) Resistance exercise with 5-min warm up and 5-min cool down (total 60 min) which included toe and heel raises, knee lifts, knee extension, seated rowing with the use of resistance band, lateral leg raises and standard squats, 20 repetitions, 2 sets,, (ii) Nutritional program which included general lecture, practical activities and group activities, and (iii) Psychosocial program (30 min) which was aimed to enhance participant social capital, 2x/wk over 3 months	Hand grip strength, kg*				
						29.0 (7.8)	29.7 (8.0)			
						Usual gait speed, m/s*				
						1.4 (0.22)	1.45 (0.24)			
						One-legged stance, sec*				
						27.3 (24.6)	29.2 (24.9)			
						TUG, sec*				
						6.04 (1.6)	5.74 (1.38)			
	Delayed intervention (*n* = 39)	74.3 (5.6)	M 29, F 10	30 (76.9)	Continued with daily activities for the initial 3 months, then participated in multifactorial intervention (as above)	Hand grip strength, kg*				
						29.8 (9.8)	31.2 (10.1)			
						Usual gait speed, m/s*				
						1.39 (0.27)	1.45 (0.28)			
						One-legged stance, sec*				
						26.6 (22.8)	33.0 (24.2)			
						TUG, sec*				
						5.99 (1.42)	5.94 (1.43)			
Serra-Prat [43], RCT						(baseline)	(follow-up at 12 months)		6	High
	Intervention (*n* = 80)	77.9 (5.0)	M 39, F 41	172 (100.0)	Physical activity (i) Aerobic exercise which included walking outdoors for 30–45 min/day, (ii) Strengthening exercises and balance exercises which included upper and lower limb strengthening, and balance and coordination for 20–25 min, total 60 min per session, at least 4x/wk over 2–3 months Nutritional activity; screened for malnutrition, and referred for assessment	Hand grip, kg		Hand grip -0.50 [-1.88, 0.88] Walking speed 0.10 [0.04, 0.16] TUG test 0.00 [-0.34, 0.34] Odds ratio—pre-frailty reversal 1.50 [0.62, 3.65]		
						15.9 (4.7)	15.6 (5.1)			
						Walking speed, m/s				
						0.9 (0.2)	1.0 (0.2)			
						TUG test, sec				
						9.3 (3.2)	8.0 (2.5)			
						Number of pre-frail who transitioned to robust, n				
						13 (out of 61)				
	Control (*n* = 92)	78.8 (4.9)	M 36, F 56		Received usual care and recommendations	Hand grip, kg				
						16.3 (4.0)	16.5 (4.4)			
						Walking speed, m/s				
						0.9 (0.2)	0.9 (0.2)			
						TUG test, sec				
						9.3 (3.5)	8.0 (2.1)			
						Number of pre-frail who transitioned to robust, n				
						11 (out of 72)				
Tan [22], RCT						(baseline)	(6 months post-baseline)	Exercise vs Control Handgrip strength 0.25 (-1.2, 1.7) Gait speed 0.18 (0.07, 0.29) 5 × STS time -0.08 (-2.05, 1.89) SPPB 0.22 (-0.48, 0.92) MoCA 0.2 (-0.77, 1.17) Exercise + Cognitive Stimulation Therapy vs Control Handgrip strength 0.18 (-1.16, 1.52) Gait speed 0.13 (0.04, 0.22) 5 × STS time 1.83 (-0.03, 3.69) SPPB 0.37 (-0.29, 1.03) MoCA 0.72 (-0.25, 1.69)	3	High
	Exercise (*n* = 26)	73.39 (5.2)	Not reported‡	173 (100.0)	Multicomponent exercise program (i) Aerobic training, (ii) Resistance training, (iii) Dual task, and (iv) Balance training, 1 h, 2x/wk over 6 months	Handgrip strength, kg*				
						Mean change (95% CI) -0.41 (-1.82, 0.99)				
						Gait speed, m/s*				
						Mean change (95% CI) 0.20 (0.10, 0.31)				
						5 × STS time, secs*				
						Mean change (95% CI) 0.88 (-0.99, 2.76)				
						SPPB, score*				
						Mean change (95% CI) 0.29 (-0.38, 0.96)				
						MoCA, score*				
						Mean change (95% CI) 0.51 (-0.40, 1.41)				
	Exercise + Cognitive Stimulation Therapy (*n* = 25)	72.56 (5.06)			Multicomponent exercise program (as per Exercise group) Cognitive stimulation therapy (games, food, current affairs, art and word association), 30 min, 2x/wk over 3 months	Handgrip strength, kg*				
						Mean change (95% CI) -0.48 (-1.76, 0.79)				
						Gait speed, m/s*				
						Mean change (95% CI) 0.15 (0.05, 0.24)				
						5 × STS time, secs*				
						Mean change (95% CI) -1.03 (-2.78, 0.72)				
						SPPB, score*				
						Mean change (95% CI) 0.44 (-0.19, 1.07)				
						MoCA, score*				
						Mean change (95% CI) 1.03 (0.12, 1.94)				
	Control (*n* = 122)	71.69 (4.99)			General health education advice	Handgrip strength, kg*				
						Mean change (95% CI) -0.66 (-1.25, -0.08)				
						Gait speed, m/s*				
						Mean change (95% CI) 0.02 (-0.04, 0.05)				
						5 × STS time, secs*				
						Mean change (95% CI) 0.80 (-0.04, 1.64)				
						SPPB, score*				
						Mean change (95% CI) 0.07 (-0.22, 0.36)				
						MoCA, score*				
						Mean change (95% CI) 0.31 (-0.15, 0.76)				
Teh [83], RCT						(baseline)	(after 24 months)	Exercise vs Control Odds ratio—pre-frailty reversal 1.13 [0.22, 5.85] Combined vs Control Odds ratio—pre-frailty reversal 4.14 [1.11, 15.42]	7	Some concerns
	Nutrition (*n* = 60)	80.0 (5.2)	Not reported‡	249 (100.0)	Nutrition education and cooking programme which included learning standardised nutrition education, cooking a recipe and sharing the cooked meal, 3 h, 1x/wk over 8 weeks	Number of pre-frail who transitioned to robust, n*				
						8				
	Exercise (*n* = 56)	79.9 (4.9)			Exercises adapted from the home-delivered Otago Exercise Program to a community group delivery format, 1 h, 1x/wk over 10 weeks	Number of pre-frail who transitioned to robust, n*				
						3				
	Combined (*n* = 70)	79.8 (5.2)			As per Nutrition and Exercise groups	Number of pre-frail who transitioned to robust, n*				
						12				
	Control (*n* = 63)	81.4 (5.2)			Social gathering with activities such as board or card games, craft, and conversation groups, 1x/wk over 10 weeks	Number of pre-frail who transitioned to robust, n*				
						3				
Tou [17], RCT						(baseline)	(after 3 months)	Handgrip strength -0.64 [-3.03, 1.75] SPPB—Chair stand score 1.23 [-1.74, 4.20] SPPB—Balance score 0.21 [-0.16, 0.58] SPPB—Gait speed score -0.16 [-0.47, 0.15] SPPB score 0.76 [-0.11, 1.63] TUG -0.88 [-2.61, 0.85]	6	Some concerns
	Intervention (*n* = 27)	72.1 (8.14)	M 2, F 25	26 (96.3)	Progressive functional power training exercises with the use of body weight and/or resistance bands (i) Lower-body power which included sit-to-stand, squats, hip extension, hip abduction, and calf raises, (ii) Upper-body power which included bicep curls, chest press, seated low row, and shoulder press, (iii) Balance and mobility exercises which included tandem balance, speed walk, mini lunges etc., 1 h, 2x/wk over 12 weeks, Received monthly health education talks on nutrition and cognition	Handgrip strength, kg				
						18.1 (3.65)	18.7 (4.52)			
						SPPB—Chair stand score				
						3.41 (0.69)	3.87 (0.34)			
						SPPB—Balance score				
						3.67 (0.68)	3.74 (0.54)			
						SPPB—Gait speed score				
						3.78 (0.58)	3.91 (0.29)			
						SPPB score				
						10.9 (1.46)	11.5 (0.73)			
						TUG, sec				
						8.92 (2.49)	8.32 (2.27)			
	Control (*n* = 30)	71.5 (8.0)	M 2, F 28	30 (100.0)	Continue with the available exercise program at the respective senior activity centers + Given exercise manual with a list of exercises + Encouraged to attend health education talks	Handgrip strength, kg				
						17.4 (3.95)	18.6 (4.81)			
						SPPB—Chair stand score				
						3.43 (1.04)	3.44 (1.09)			
						SPPB—Balance score				
						3.73 (0.58)	3.59 (0.8)			
						SPPB—Gait speed score				
						3.73 (0.58)	3.7 (0.67)			
						SPPB score				
						10.9 (1.65)	10.8 (2.0)			
						TUG, sec				
						9.22 (3.27)	9.5 (4.13)			
Yu [44], RCT						(baseline)	(after 3 months)	Grip strength 0.7 (-0.20, 1.60) Muscle endurance—Chair stand 3.80 (2.63, 4.97) Gait speed -0.20 (-0.57, 0.17) Executive function—FAB 2.00 (0.96, 3.04) Life statisfaction 0.10 (-0.28, 0.48) Odds ratio—pre-frailty reversal 300.0 [37.5, 2400.27]	8	Some concerns
	Intervention (*n* = 66)	62.2†	M 15, F 112	127 (100.0)	Multicomponent frailty prevention program (i) Exercise which included warm up and cool down (range of motion exercises for different joints of the body), 25-min aerobic exercise (circuit training comprised of exercises such as marching on the spot, squatting, stepping up and down etc.), and 25-min resistance training (knee extension, chest stretching, squatting etc.) (ii) Cognitive training and board game activities for 1 h, 2x/wk over 12 weeks	Grip strength, kg				
						23.1 (6.3)	23.8 (6.3)			
						Muscle endurance—chair stand, secs				
						11.8 (4.6)	9.4 (2.8)			
						Gait speed, secs				
						6.4 (1.3)	6.4 (1.0)			
						Executive function—FAB, score				
						14.5 (2.7)	16.5 (2.1)			
						Life satisfaction, score				
						7.1 (1.2)	7.6 (1.1)			
						Number of pre-frail who transitioned to robust, n				
						55				
	Control (*n* = 61)				Participants were put on a waiting-list	Grip strength, kg				
						22.3 (6.2)	22.2 (5.6)			
						Muscle endurance—chair stand, secs				
						12.2 (3.6)	13.5 (4.6)			
						Gait speed, secs				
						6.6 (1.1)	6.7 (1.1)			
						Executive function—FAB, score				
						14.2 (2.8)	14.2 (3.1)			
						Life satisfaction, score				
						6.9 (1.2)	7.3 (1.4)			
						Number of pre-frail who transitioned to robust, n				
						1				
Zech [84], RCT						(baseline)	(6 months post-baseline)	Strength training vs Control Balance 0.20 (-0.54, 0.94) Gait 0.10 (-0.32, 0.52) Chair rise -0.20 (-0.92, 0.52) SPPB 0.10 (-1.32, 1.52) Power training vs Control Balance 1.0 (0.24, 1.76) Gait 0.0 (-0.45, 0.45) Chair rise 0.40 (-0.35, 1.15) SPPB 1.30 (-0.17, 2.77)	8	Some concerns
	Strength training (*n* = 18)	77.8 (6.1)	Not reported‡	18 (100.0)	(i) 5-min of warm-up (walking exercises), (ii) 20-min of balance exercises performed on stable ground, mats and wobble boards with ball-catching exercises, and (iii) 25-min of muscle strength exercises using resistance training machine, performed the concentric and eccentric contractions with average velocity (2–3 secs) 2x/wk over 12 weeks	SPPB—Balance, points*				
						2.5 (1.0)	2.6 (1.2)			
						SPPB—Gait, points*				
						3.5 (0.8)	3.6 (0.9)			
						SPPB—Chair rise, points*				
						2.8 (1.2)	2.6 (1.1)			
						SPPB, points*				
						8.8 (2.4)	8.9 (2.3)			
	Power training (*n* = 16)	77.4 (6.2)	Not reported‡	16 (100.0)	(i) 5-min of warm-up (walking exercises), (ii) 20-min of balance exercises (as per strength training group), and (iii) 25-min of muscle power exercises using resistance training machine, instructed to move as rapidly as possible during the concentric phase of each repetition and then slowly during the eccentric phase (approximately 2–3 secs), 2x/wk over 12 weeks	SPPB—Balance, points*				
						2.3 (1.2)	3.2 (1.0)			
						SPPB—Gait, points*				
						3.8 (0.5)	3.8 (0.4)			
						SPPB—Chair rise, points*				
						2.9 (1.1)	3.3 (0.8)			
						SPPB, points*				
						9.0 (2.1)	10.3 (1.5)			
	Controls (*n* = 20)	75.9 (7.8)	Not reported‡	20 (100.0)	Instructed to maintain their usual physical activity	SPPB—Balance, points*				
						3.1 (1.2)	3.0 (1.2)			
						SPPB—Gait, points*				
						3.9 (0.4)	3.9 (0.2)			
						SPPB—Chair rise, points*				
						3.2 (1.0)	3.2 (1.2)			
						SPPB, points*				
						10.2 (2.1)	10.2 (2.1)			

Abbreviation(s): *Values which gave worst outcome (mean difference) were extracted; when measures were reported over dominant/non-dominant side or more than 1 time-point

^†^Mean and/or standard deviation not reported

^‡^Gender not reported. *RCT *Randomised controlled trial, *HRQOL *Health-Related Quality of Life based on the Short Form-36 (score out of 100), with higher scores indicating better quality of life, *SPPB *Short Physical Performance Battery (score out of 12), with higher scores indicating greater physical function, *TUG *Timed up and go, *MMSE *Mini Mental State Examination (score out of 30), with higher scores indicating better cognition, *MoCA *Montreal Cognitive Assessment (score out of 30), with higher scores indicating better cognition, *RBANS *Repeatable Battery for the Assessment of Neuropsychological Status (score out of 160), with higher index score indicating better cognitive performance, *FAB *Frontal Assessment Battery (score out of 18), with higher scores indicating better performance in executive functions

### Study characteristics

**Fig. 2:**
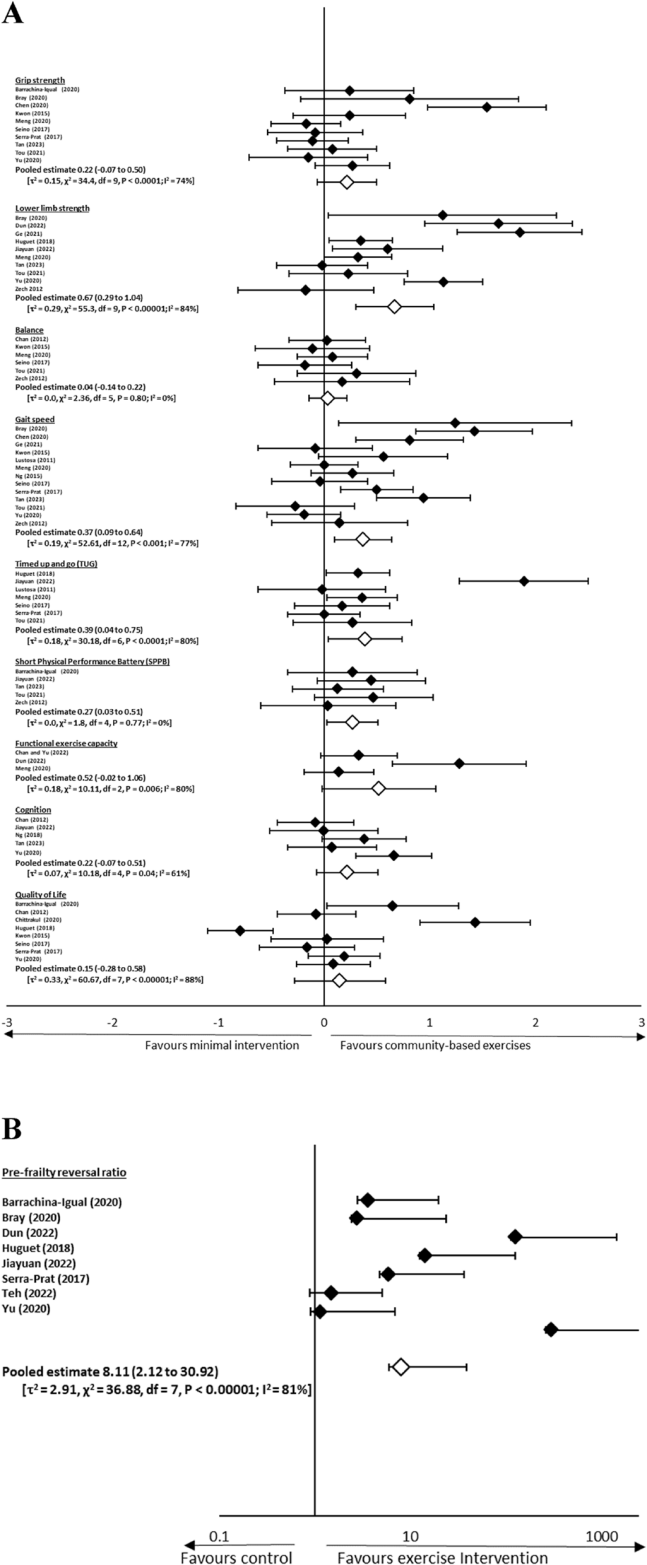
**A** Forest plot (standardised mean difference and 95% CI), and (**B)** (odds ratio and 95% CI) of outcome measures in randomized controlled trials. Pooled estimates of subgroup outcome measures are indicated by empty symbols

**Fig. 3:**
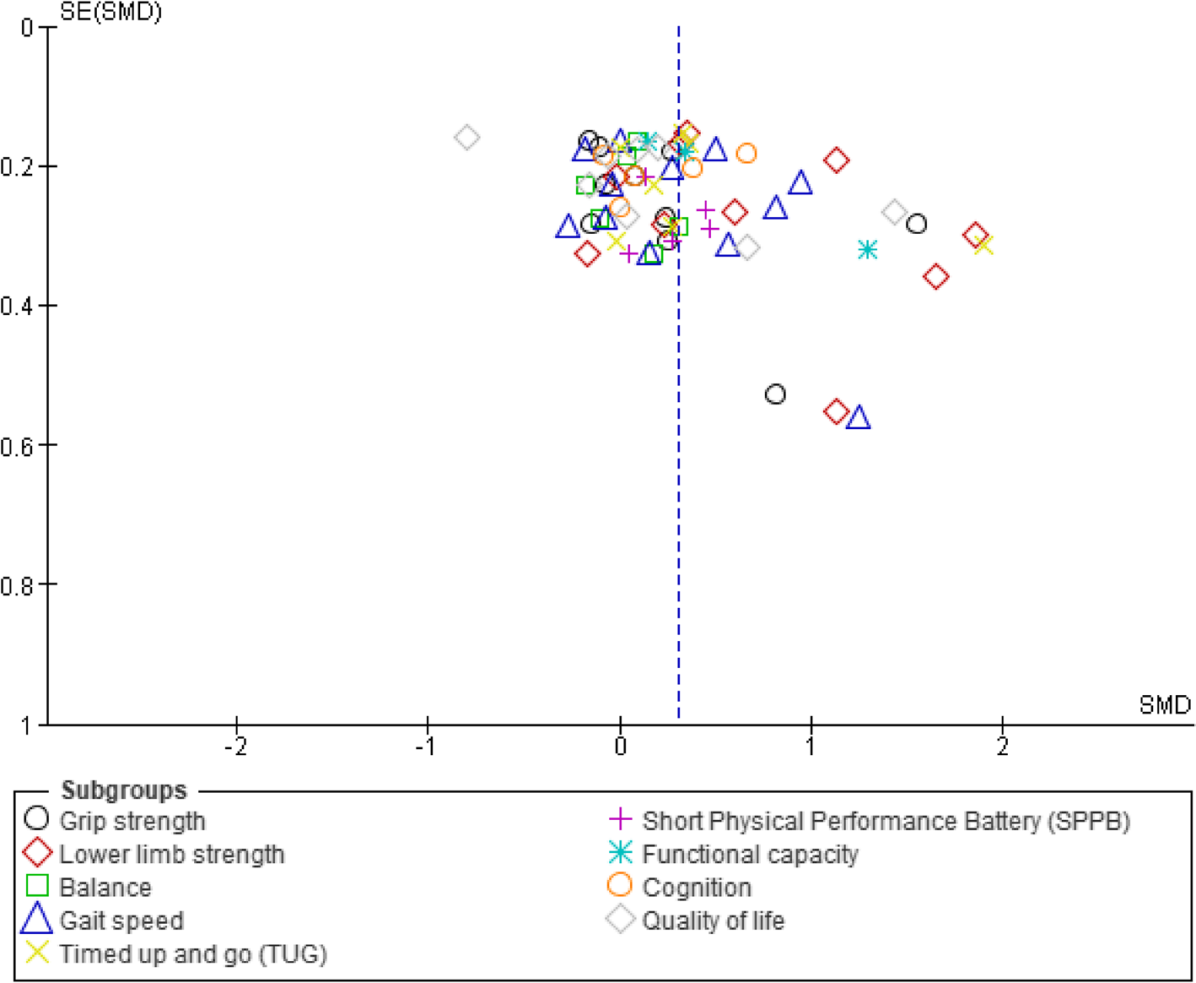
Funnel plot of standardised mean difference (SMD) against standard error of SMD in outcome measures

Twenty-two randomized controlled trials (900 participants in the experimental group and 1015 participants in the control group), which had data for physical measures, cognition, frailty status and/or quality of life were available for pooling (Fig. 2). The criteria used for determining pre-frailty across the trials included Fried’s frailty criteria [10, 11, 14, 17, 21, 43, 76–80, 84], FRAIL questionnaire [22, 44, 74, 83], Cardiovascular Health Study criteria [23, 81], Frailty phenotype [26], Kaigo-Yobo Checklist [82], Chinese Canadian Study of Health and Aging Clinical Frailty Scale Telephone Version [73], and was not mentioned in one of the trials [75]. Ten trials evaluated the effects of multi-component exercise [10, 17, 21, 22, 26, 44, 73, 75, 80, 84], 4 trials on multi-component exercise with nutrition [43, 77, 78, 83], 2 trials on multi-component exercise with nutrition and cognitive [81, 85], 2 trials on TaiChi [11, 76], 1 trial on strengthening exercises with nutrition [82], 1 trial on strengthening exercises [79], 1 trial on elastic band [14], and 1 trial on stepping exercises [74]. Six trials were found to have high risk of bias [22, 26, 43, 74, 77, 85], whilst there was some concerns in 15 trials [11, 14, 17, 21, 44, 73, 75, 76, 78–84], and low risk of bias in 1 trial [10]. Evidence of symmetry was visually confirmed in the funnel plot (Fig. 3). A symmetrical distribution in the studies about the combined effect size was observed in Fig. 3.

### Grip strength

Ten trials (378 participants in the experimental group and 482 participants in the control group) had data for grip strength [14, 17, 21, 22, 26, 43, 44, 78, 80, 82]. Data were pooled using a random-effects model; there was a non-significant pooled standardized mean difference in grip strength (0.22, 95% CI -0.07 to 0.50, *P* > 0.05) between exercise and minimal intervention, with a high level of heterogeneity (*I*^*2*^ = 74%, τ^2^ = 0.15, χ^2^ = 34.44, df = 9, *P* = 0.0001) (Fig. 2).

### Lower limb strength

Ten trials (384 participants in the experimental group and 482 participants in the control group) had data for lower limb strength [10, 11, 17, 22, 26, 44, 76, 77, 80, 84]. Data were pooled using a random-effects model; there was a significant pooled standardized mean difference in lower limb strength (0.67, 95% CI 0.29 to 1.04, *P* < 0.0001) between exercise and minimal intervention, with a high level of heterogeneity (*I*^*2*^ = 84%, τ^2^ = 0.29, χ^2^ = 55.3, df = 9, *P* < 0.00001) (Fig. 2). Whilst there were no apparent outliers, we performed post-hoc sensitivity analysis by removing 3 RCTS with high risk of bias [22, 26, 77]. The pooled effect size for lower limb strength (7RCTs, 265 participants in the experimental group and 264 participants in the control group) remained significant (0.79, 95% CI 0.29 to 1.29, *P* = 0.002), with a high level of heterogeneity (*I*^*2*^ = 86%, τ^2^ = 0.38, χ^2^ = 42.07, df = 6, *P* < 0.00001).

### Balance

Six trials (233 participants in the experimental group and 248 participants in the control group) had data for balance [17, 73, 78, 80, 82, 84]. Data were pooled using a random-effects model; there was a non-significant pooled standardized mean difference in balance (0.04, 95% CI -0.14 to 0.22, *P* = 0.69) between exercise and minimal intervention, with a low level of heterogeneity (*I*^*2*^ = 0%, τ^2^ = 0.0, χ^2^ = 2.36, df = 5, *P* = 0.80) (Fig. 2).

### Gait speed

Thirteen trials (484 participants in the experimental group and 581 participants in the control group) had data for gait speed [14, 17, 22, 26, 43, 44, 76, 78–82, 84]. Data were pooled using a random-effects model; there was a significant pooled standardized mean difference in gait speed (0.37, 95% CI 0.09 to 0.64, *P* = 0.009) between exercise and minimal intervention, with a high level of heterogeneity (*I*^*2*^ = 77%, τ^2^ = 0.19, χ^2^ = 52.61, df = 12, *P* < 0.001) (Fig. 2). Similarly, in the absence of apparent outliers, we proceeded with post-hoc sensitivity analysis by removing 3 RCTS with high risk of bias [22, 26, 43]. The pooled effect size for gait speed (10 RCTs, 389 participants in the experimental group and 379 participants in the control group) was not statistically significant (0.25, 95% CI -0.06 to 0.55, *P* = 0.11), with a high level of heterogeneity (*I*^*2*^ = 76%, τ^2^ = 0.18, χ^2^ = 37.5, df = 9, *P* < 0.001).

### Timed up and go (TUG)

Seven trials (343 participants in the experimental group and 344 participants in the control group) had data for TUG [11, 17, 43, 77, 79, 80, 82]. Data were pooled using a random-effects model; there was a significant pooled standardized mean difference in TUG (0.39, 95% CI 0.04 to 0.75, *P* < 0.0001) between exercise and minimal intervention. Due to the high level of heterogeneity (*I*^*2*^ = 80%, τ^2^ = 0.18, χ^2^ = 30.18, df = 6, *P* < 0.0001) (Fig. 2), we performed post-hoc sensitivity analyses by removing an outlier [11]. However, the pooled standardized mean difference (6 RCTs, 313 participants in the experimental group and 314 participants in the control group) remained significant (0.21, 95% CI 0.06 to 0.37, *P* = 0.008) with a low level of heterogeneity (*I*^*2*^ = 0%, τ^2^ = 0.0, χ^2^ = 3.38, df = 5, *P* = 0.64). But when we repeated the analysis by removing 2 RCTS with high risk of bias [43, 77], the pooled effect size for TUG (5 RCTs, 197 participants in the experimental group and 184 participants in the control group) was not statistically significant (0.52, 95% CI -0.04 to 1.07, *P* = 0.07), with a high level of heterogeneity (*I*^*2*^ = 84%, τ^2^ = 0.33, χ^2^ = 25.64, df = 4, *P* < 0.0001).

### Short physical performance battery (SPPB)

Five trials (120 participants in the experimental group and 219 participants in the control group) had data for SPPB [11, 17, 21, 22, 84]. Data were pooled using a random-effects model; there was a significant pooled standardized mean difference in SPPB (0.27, 95% CI 0.03 to 0.51, *P* = 0.03) between exercise and minimal intervention, with a low level of heterogeneity (*I*^*2*^ = 0%, τ^2^ = 0.0, χ^2^ = 1.8, df = 4, *P* = 0.77) (Fig. 2). Similarly, we performed post-hoc sensitivity analysis albeit there were no apparent outliers. We removed 1 RCT with high risk of bias [22], but the significance in pooled effect size for SPPB (4 RCTs, 94 participants in the experimental group and 97 participants in the control group) persisted (0.33, 95% CI 0.04 to 0.62, *P* = 0.02) with a low level of heterogeneity (*I*^*2*^ = 0%, τ^2^ = 0.0, χ^2^ = 1.23, df = 3, *P* = 0.75).

### Functional capacity

Three trials (159 participants in the experimental group and 159 participants in the control group) had data for functional capacity [10, 74, 80]. Data were pooled using a random-effects model; there was a non-significant pooled standardized mean difference in functional capacity (0.52, 95% CI -0.02 to 1.06, *P* = 0.06) between exercise and minimal intervention, with a high level of heterogeneity (*I*^*2*^ = 80%, τ^2^ = 0.18, χ^2^ = 10.11, df = 2, *P* = 0.006) (Fig. 2).

### Cognition

Five trials (225 participants in the experimental group and 325 participants in the control group) had data for cognition [11, 22, 44, 73, 85]. Data were pooled using a random-effects model; there was a non-significant pooled standardized mean difference in cognition (0.22, 95% CI -0.07 to 0.51, *P* = 0.14) between exercise and minimal intervention, with a moderate level of heterogeneity (*I*^*2*^ = 61%, τ^2^ = 0.07, χ^2^ = 10.18, df = 4, *P* = 0.04) (Fig. 2).

### Quality of life

Eight trials (390 participants in the experimental group and 406 participants in the control group) had data for quality of life [21, 43, 44, 73, 75, 77, 78, 82]. Data were pooled using a random-effects model; there was a non-significant pooled standardized mean difference in quality of life (0.15, 95% CI -0.28 to 0.58, *P* = 0.50) between exercise and minimal intervention, with a high level of heterogeneity (*I*^*2*^ = 88%, τ^2^ = 0.33, χ^2^ = 60.67, df = 7, *P* < 0.00001) (Fig. 2).

### Reversal of frailty status

Eight trials (351 participants in the experimental group and 363 participants in the control group) had data for the proportion of frailty status [10, 11, 21, 26, 43, 44, 77, 83]. Data were pooled using a random-effects model; pre-frail older adults who received community-based exercises were more likely to reverse from pre-frailty to robust state (OR = 8.11, 95% CI 2.12 to 30.92, *P* = 0.002), when compared to those who received minimal intervention. Due to the high level of heterogeneity (*I*^*2*^ = 81%, τ^2^ = 2.91, χ^2^ = 36.88, df = 7, *P* < 0.00001), we performed post-hoc sensitivity analysis, that is, we removed two outliers [10, 44]. However, the pooled odds ratio (6 RCTs, 263 participants in the experimental group and 281 participants in the control group) remained significant (OR = 2.74, 95% CI 1.36 to 5.51, *P* = 0.005) with a low level of heterogeneity (*I*^*2*^ = 23%, τ^2^ = 0.18, χ^2^ = 6.48, df = 5, *P* = 0.26). Thereafter, we repeated the analysis by removing 3 RCTS with high risk of bias [26, 43, 77], the pooled odds ratio of pre-frailty reversal (5 RCTs, 197 participants in the experimental group and 195 participants in the control group) remained persistently significant (OR = 14.01, 95% CI 1.89 to 103.58, *P* = 0.01), with a high level of heterogeneity (*I*^*2*^ = 84%, τ^2^ = 4.32, χ^2^ = 24.88, df = 4, *P* < 0.0001).

### Parameters of community-based exercise as predictors of the effect size measures

The most commonly used parameters were 60-min duration [11, 17, 22, 43, 44, 73–76, 78, 79, 83], 3 sessions per week [10, 14, 26, 73–77, 79, 80] over a time span of 12 weeks [10, 17, 21, 26, 44, 73–75, 78, 80–82, 84, 85]. Our multivariable meta-regression analyses identified frequency (*Beta* = 0.6, 95% CI 0.084 to 1.115, *P* = 0.028) as an independent predictor of the effect size for gait speed amongst older adults with pre-frailty (Table 2). In other words, increased frequency per week was associated with greater effect size for gait speed. The model correctly predicted 56.6% of the variability in the effect size for gait speed. The normality in the distribution of residuals and homoscedasticity in the scatterplot were visualised. Based on the variance inflation factor value, there was no evidence of multicollinearity.

**Table 2:** Predictors of the effect size for gait speed in pre-frail older adults

Dependent variables	Independent variables	Unstandardized coefficients		95% CI for β		Standardized coefficients	*t*-Value	Significance
		*B*	SE	Lower	Upper	Beta		
Standardized mean difference in gait speed	Frequency (times/week)	0.60	0.224	0.084	1.115	0.674	2.683	0.028
	Time span (weeks)	0.039	0.036	-0.044	0.122	0.274	1.094	0.306
	Duration (mins)	-0.013	0.008	-0.031	0.006	-0.369	-1.577	0.154
	Constant	-0.64	0.988	-2.919	1.639		-0.648	0.535

*B* regression coefficient, *SE* standard error of, *B* Beta, Beta coefficient, *t* t-statistics, *95% CI* 95% confidence interval for regression coefficient

### GRADE

The strength of evidence is illustrated in Table 3 according to the GRADE criteria with an overall certainty of evidence ranging from very low to moderate.

**Table 3:** Quality of the evidence (GRADE) for SMD in significant outcome measures

Quality assessment						Summary of findings		
No of studies (Design)	Risk of bias	Publication bias	Imprecision	Inconsistency	Indirectness	No of participants (Intervention/Control)	Pooled SMD (95% CI)	Quality of evidence
Lower limb strength								
10 RCTs	Detected – failure to conceal allocation and failure to blind	Not detected	No serious imprecision	High *I*^2^ value, but similarity in point estimates after post-hoc sensitivity analysis	No serious indirectness	384/482	0.67 (0.29, 1.04)	⊕⊕⊕〇 **Moderate**
Gait speed								
12 RCTs	Detected – failure to conceal allocation and failure to blind	Not detected	Confidence interval crosses decision threshold	Inconsistent results due to high *I*^2^ value	No serious indirectness	451/548	0.27 (0.03, 0.52)	⊕〇〇〇 **Very Low**
Timed-up-and-go								
7 RCTs	Detected – failure to conceal allocation and failure to blind	Not detected	Confidence interval crosses decision threshold	Inconsistent results due to lack of overlapping of confidence intervals, and high *I*^2^ value	No serious indirectness	343/344	0.39 (0.04, 0.75)	⊕〇〇〇 **Very Low**
Short Physical Performance Battery								
5 RCTs	Detected – failure to conceal allocation and failure to blind	Not detected	Confidence interval crosses decision threshold	No serious inconsistency	No serious indirectness	120/219	0.27 (0.03, 0.51)	⊕⊕〇〇 **Low**
Pre-frailty reversal ratio								
8 RCTs	Detected – failure to conceal allocation and failure to blind	Not detected	No serious imprecision	Inconsistent results due to high *I*^2^ value	No serious indirectness	351/363	8.11 (2.12, 30.92)	⊕⊕〇〇 **Low**

## Discussion

This systematic review has synthesized the evidence for the role of community-based exercises in improving lower limb strength and function (SMD = 0.27–0.67, *P* < 0.05) when compared to minimal intervention in pre-frail older adults (Supplementary Fig. 2Ai). In addition, community-based exercises is superior to minimal intervention in reversing pre-frailty to healthy state amongst them. The frequency, that is, the number of community-based exercise sessions per week, may be a predictor of the effect size of gait speed in pre-frail older adults. These findings have implications on the implementation of public health intervention such as community-based exercises targeted at pre-frailty.

We did not find a significant pooled SMD in grip strength between pre-frail older adults who have received community-based exercises and those who have received minimal intervention. In comparison with a recent review by Liu and co-workers (2022) [86], they have reported significant pooled MD in grip strength, that is, pooled MD of 1.36 from 4 studies which investigated exercise only, and pooled MD of 2.71 from 2 studies which investigated the effects of exercise with nutrition (Fig. 2 therein, p1431.e5) [86]. We propose that the inconsistency in findings between reviews may be explained by a few methodologically plausible reasons, that is, the different types of dynamometer that have been used across the studies in our review [21, 26, 44], and the different methods of assessing grip strength with variation of the protocol or body position [87, 88]. Perhaps a greater consistency in methodology in future studies may yield further insight on this. Having said this, we have calculated the SMD value which would have accounted for the variation in spread of data due to the different testing methods and exercise protocols.

Our review has revealed significantly moderate effect size in lower limb strength when comparing pre-frail older adults who received pre-frailty intervention compared to minimal intervention (SMD = 0.67). During our post-hoc subgroup analyses of trials which used timed 5-times sit-to-stand test (*n* = 4) [22, 26, 44, 77], the significance in pooled lower limb strength remained (SMD = 0.58, *p* = 0.04). This corresponds to a reduction by 2.25 secs (Supplementary Fig. 4Aii), which concurs with the minimal clinically important difference, that is 0.5 to 1.7 secs, as reported by a previous study [89]. When we analysed trials which used 30 s chair rise stand test (*n* = 3) [10, 11, 76], the significance in pooled lower limb strength persisted as well (SMD = 1.35, *p* = 0.001), and this is borne out to be approximately 4 repetitions. On the contrary, Liu and co-workers (2022) reported a lack of significance in pooled mean difference [86]. This discordance may plausibly be due to the trials included during analysis. For example, we included trials which recruited mostly community-dwelling older adults with pre-frailty [10, 11, 76], whereas Liu and co-workers (2022) [86] included trials which recruited hospitalized pre-frail older adults [56], pre-frail older adults from residential living centres [90], pre-frail elderly women who visited the sport training centre [91], and community-dwelling pre-frail elderly people [76]. Future reviews may include more specific inclusion criteria to enhance comparison between studies, and to better understand the target population being studied.

We have found a significantly small pooled effect size in gait speed (SMD = 0.37) with more precise estimate, and this correlates with a reduction in time taken by approximately 0.16 s to complete the gait speed test. Similarly, Liu and co-workers (2022) [86] have also reported a significant pooled effect size in gait speed. Conversely, they have reported a higher effect size (SMD = 1.06) with less precise estimate. This differential in effect size could be attributed to the difference in method of data extraction, that is, we have extracted change score whilst Liu and co-workers (2022) extracted follow-up scores [92]. Secondly, Liu and co-workers (2022) included 4 trials with exercise-only intervention [14, 44, 76, 79] in their review during analysis of pooled SMD in gait speed (Supplementary Fig. 6 therein, p1431e.13). In contrast, we included 13 trials with diverse exercise protocols during analysis. Interestingly, our post-hoc subgroup analysis which looked at 6 trials with multi-component exercises yielded a lack of significance in pooled SMD in gait speed (Supplementary Fig. 3). Overall, we believe that our estimated effect size in gait speed herein is considered conservative in view of the larger number of studies included in our analysis. Future reviews may consider the extraction of change score, instead of follow-up scores to yield a more precise estimate.

Our review has unveiled significantly small effect size in timed up-and-go (SMD = 0.39), and this parallels with a reduction in timing by 0.73 secs. However, this is less than the minimum detectable change of 2.08 secs reported by a previous study on community dwelling adults aged 50 and above [93]. Our estimated minimal clinically important difference for timed up-and-go worked out to be 0.41 secs [94]. We are unaware of any available minimal clinically important difference values for timed up-and-go in frail or pre-frail older adults within the literature for comparison. In contrast to our finding, Liu and co-workers (2022) have reported a lack of significance in pooled effect size in timed up-and-go. The lack of significance remained based on their subgroup analyses of trials which investigated the effect of exercise only, and trials which investigated the effect of exercise with nutrition (Supplementary Fig. 9 therein, p1431.e16). We were unable to replicate the aforementioned subgroup analyses on a post-hoc basis due to the diverse exercise protocols. For similar reason, we believe that the discrepancy in our findings may be attributed to the difference in trials included during analaysis.

We have found a significantly pooled effect size in SPPB, and this concurs with the finding by Liu and co-workers (2022) [86]. Our pooled effect size in SPPB yielded a SMD of 0.27, and this is borne out to be 0.45 point, which is considered clinically significant [95]. In addition, we estimated that the minimal clinically important difference in SPPB is 0.83 point [94]. In contrast, Liu and co-workers (2022) have reported a much larger overall pooled mean difference in SPPB of 0.81 point; pooled mean difference of 1.02 points from 4 studies which investigated exercise only, and pooled mean difference of 1.2 points from 1 study which investigated exercise with nutrition (Fig. 1 therein, p1431.e4). For similar reason, it is plausible that the difference in magnitude of effect size may be due to the difference in method of data extraction. SPPB, which is a composite measure of balance, gait speed and lower limb strength, has been reported to be a protective frailty factor and can be monitored in pre-frail older adults [96]. These corroborate the use of SPPB as a tool, at least in part, in evaluating the effectiveness of community pre-frailty intervention.

By inference of a recent study which has reported a lack of significant change in balance among retirees who were aged 60 years and above after participation in a 3-month community-based physical activity with fall prevention program [97], it is conceivable that detecting a significant change in balance with community-based exercises among pre-frail older adults may be just as challenging as in our review. Having said these, it is noteworthy that studies which used SPPB balance score [17, 84] had consistently larger effect size estimates than studies which used one-legged stance test [73, 78, 80, 82]. This suggests that SPPB balance score which assesses the ability to assume normal, semi-tandem and tandem stance for 10 s, may be more sensitive in detecting changes when compared to the timed one-legged stance test. Interestingly, some of the included trials in this analysis did not include balance exercises in their pre-frailty program [80, 82]. This may highlight the importance of incorporating balance exercises in the community pre-frailty intervention. Future studies may consider the use of SPPB balance score, instead of the single leg stance test in evaluating balance performance.

Notwithstanding the inclusion of aerobic exercises in the pre-frailty intervention across the included trials [10, 74, 80], there is a lack of significance in the pooled effect size for functional exercise capacity. To our knowledge, we are unaware of reviews which have investigated the effect of community-based exercise on functional exercise capacity in pre-frail older adults. We believe that we could have yielded a different result if there were more trials which had incorporated multi-component exercises in their protocol included in our analysis, that is, elements of resistance, aerobic, balance and flexibility training, to augment the effect on improving functional exercise capacity [98]. Our finding may also be explained by other reasons, that is, the pre-frail older adult participants were likely to be working at the limit of their physical capacity to carry out activities of daily living [99]. Lastly, when interpreting the result from a previous study [100], the training effect of cycling exercise [80] or stepping exercise [10, 74] may be inadequate to improve functional exercise capacity in pre-frail older adults. Future studies may consider multi-component exercise and the inclusion of outdoor or treadmill walking as part of the exercise protocol. Nonetheless, our result should be interpreted with caution given the relatively low number of included trials and reduced statistical power to detect difference in pooled functional exercise capacity. Hence, this merit further investigation.

We have found a lack of significance in pooled cognitive performance, and this finding did not agree with a previous review by Racey and co-workers (2021) [19]. This discrepancy in conclusion may be ascribed to the difference in method of including trials in the meta-analysis. For example, some of the trials were included more than once in their meta-analysis (Fig. 3B therein, pE740) which may have overstated the precision of their results [19]. Another plausible reason could be attributed to the diverse clinical outcomes used to measure different cognition domains across our included trials [11, 22, 44, 73, 85]. Interestingly, the removal of trials which used the Mini-Mental State Examination during post-hoc subgroup analysis uncovered a significant effect (SMD = 0.39, 0.06 to 0.72, *P* = 0.02) (Supplementary Fig. 5). Based on the neuroanatomical correlates of the cognitive measures, that is, Frontal Assessment Battery [101], Repeatable Battery for the Assessment of Neuropsychological Status [102] and Montreal of Cognitive Assessment [103], it is appealing to consider that exercise may exert its effect, at least in part, through pathways involving the pre-frontal, medial temporal and/or subcortical area respectively. Further studies are warranted to support this assertion.

Despite the positive association reported between physical activity and quality of life [104], the enhancement in quality of life by pre-frailty intervention was not observed in our review. Furthermore, our finding did not concur with previous reviews [19, 86]. This may be attributed to the different methodologies used such as method of data extraction [86] and inclusion of trials during meta-analysis [19]. It is also conceivable that the lack of significance in pooled quality of life amongst community-dwelling pre-frail older adults herein may reflect the multidimensional construct of quality of life, which may be influenced by a plethora of factors such as financial resources, health and meaning in life [105]. This merits further investigation.

Albeit the scarcity of information on pooled pre-frailty reversal odds ratio, our review has revealed that the pooled odds of reversal from pre-frailty to robust state is about 3 times amongst the older adults who received community-based exercises, when compared to those who received minimal intervention. This finding concurs with other trials [26, 106], which has demonstrated similar result. Based on a proposed method to derive the number needed to treat [52], we estimated that 20 pre-frail older adults would be required to participate in community-based exercises in order for one additional pre-frail older adult to achieve healthy robust state. In comparison to findings from one of the included trials [10], we believe that our estimated number needed to treat is considered conservative based on the diverse pre-frailty intervention across our included trials. Nevertheless, our findings have implications on public health policy, that is, it underscores the benefit of public health intervention such as pre-frailty intervention in altering frailty trajectory at the population level [22]. But this would call for recommended actions by both healthcare providers and policy makers. For example, healthcare providers could consider implementing more community-based exercise programs [107], whilst policymakers could consider integrating such programs into mainstream care for the pre-frail aging population [22].

By inference of previous studies [10, 22, 54], it is tempting to speculate that exercise intervention modifies the risk factors of frailty such as reduced walking speed by altering the body composition and immune profile. For example, the reversal of pre-frailty was reportedly associated with reduced body fat mass, increased fat-free mass and improved fitness [10]. Similarly, Tan and co-workers (2023) have also reported an improved appendicular skeletal muscle index after 3–6 months of exercise with or without cognitive stimulation therapy amongst pre-frail older adults [22]. Other proposed mechanisms include the reduction in inflammatory biomarkers such as interleukin-6 and C-reactive protein after a 6-month exercise training amongst older adults [54]. From a social psychological perspective, the benefits of regular participation in community-based exercise events may be attributed to the participants’ positive and rewarding social behaviours and experiences such as subjective enjoyment and energy level [108]. These may be mediated by a reduction in feelings of fatigue and cortisol level [109]. Nonetheless, these mechanisms merit further studies for validation.

Our review of the literature revealed variability in the temporal parameters of community-based exercise, and that it is uncertain how community-based pre-frailty intervention can be rolled out to optimize clinical benefits at the population level. Thus far, a previous study has identified weekly frequency as one of the predictors of SPPB in pre-frail and frail older adults (Table 5 therein, p11) [110]. Similarly, we have identified herein that the frequency (number of sessions per week) as a significant predictor of the effect size for gait speed. However, this predictor was not significant when univariable regression analysis was performed (*P* > 0.05). We believe that further studies in this area would elucidate further insights on the predictive potential of pre-frailty intervention parameters on the clinical outcome.

### Limitations

One of the challenges encountered during this review included the variability in the pre-frailty intervention across the included trials. However, this was overcome with the use of random-effects models a priori. Secondly, different outcome measures were used across the included trials to measure the same construct. Conversely, we expressed our pooled results in units of standard deviation, that is, standardized mean difference to circumvent this issue. Thirdly, we included trials with a mix of pre-frail and frail older adults. Nevertheless, we ran post-hoc sensitivity analyses by excluding trials which included frail older adults, and the results were consistent in most of the outcome measures. Lastly, there were high risk of bias in 6 out of 22 included RCTs. Our post-hoc sensitivity analyses revealed persistently statistically significant pooled results for lower limb strength, but not for gait speed and TUG after removing RCTs with high risk of bias, hence our data should be interpreted with caution.

## Conclusion

In conclusion, this review highlights that community-based exercises is superior to minimal intervention for improving physical function and health in older adults with pre-frailty. The frequency of exercise sessions per week may influence the effect size for gait speed amongst pre-frail older adults. Further research works are warranted to investigate responsive outcome measures and optimal parameters of community-based exercises for the community-dwelling pre-frail older adults.

### What is already known

- ▪ Pre-frailty poses a large socioeconomic burden and it affects the older adults.
- ▪ There is conflicting evidence on the effectiveness of community-based exercises in improving clinical outcomes amongst older adults with pre-frailty.

### What are the new findings

- ▪ Community-based exercise is superior to minimal intervention in improving physical function such as lower limb strength and gait speed in older adults with pre-frailty.
- ▪ The odds of reversing pre-frailty to robust state is about 3 times amongst those who received community-based exercises, when compared to minimal intervention. Out of 20 pre-frail older adults who participate in community-based exercises, one is expected to achieve healthy robust state who would not otherwise have done so.
- ▪ The frequency of exercise sessions per week may influence the effect size for gait speed in older adults with pre-frailty.

### Supplementary Information

Supplementary Material 1.

Supplementary Material 2.

Supplementary Material 3.

Supplementary Material 4.

Supplementary Material 5.

Supplementary Material 6.

Supplementary Material 7.

Supplementary Material 8.

## Data Availability

The datasets generated and/or analysed during the current study are available in Table 1 and Supplementary Figs. 2–6 herein. Further data are available from the corresponding author on reasonable request.
