# Supplementary material for: Community-based exercises improve health status in pre-frail older adults: A systematic review with meta-analysis
Source: BMC Geriatr. 2024 Jul 10;24:589. doi: 10.1186/s12877-024-05150-7 (PMC11234756; doi:10.1186/s12877-024-05150-7)
Supplement: Supplementary file 1 — Supplementary Material 1. [file 12877_2024_5150_MOESM1_ESM.docx]

**Supplementary 1:** Search strategy

**1. MEDLINE Count**

pre-frail* 1,594

community* 952.039

(pre-frail*) AND (community*) 625

Applied filter “Randomized Controlled Trial” 48

**2. CINAHL Count**

pre-frail* 627 [#1]

“Randomized Controlled Trial” 17,861[#2]

#1 AND #2 55

**3. Google Scholar Count**

pre-frail 13,400

community-based 952.039

group exercise 7,460,000

physical activity 6,340,000

randomized controlled trial 2,780,000

“pre-frail” AND “community-based” AND “group exercise”

AND “physical activity” AND “randomized controlled trial” 301

“pre-frail” AND “community-based” AND “group exercise”

AND “physical activity” AND “randomized controlled trial” NOT “protein” 132

**4. Web of Science Count**

pre-frail* 1,231

community* 1,570,763

ALL=(randomized controlled trial) 479,364

(#1 AND #2) AND ALL=#3 58
