# Supplementary material for: Community-based exercises improve health status in pre-frail older adults: A systematic review with meta-analysis
Source: BMC Geriatr. 2024 Jul 10;24:589. doi: 10.1186/s12877-024-05150-7 (PMC11234756; doi:10.1186/s12877-024-05150-7)

**Supplementary 2:** Forest plots for all outcome measures in SMD, MD and OR.

**Ai.** Pooled SMD for measures with continuous data


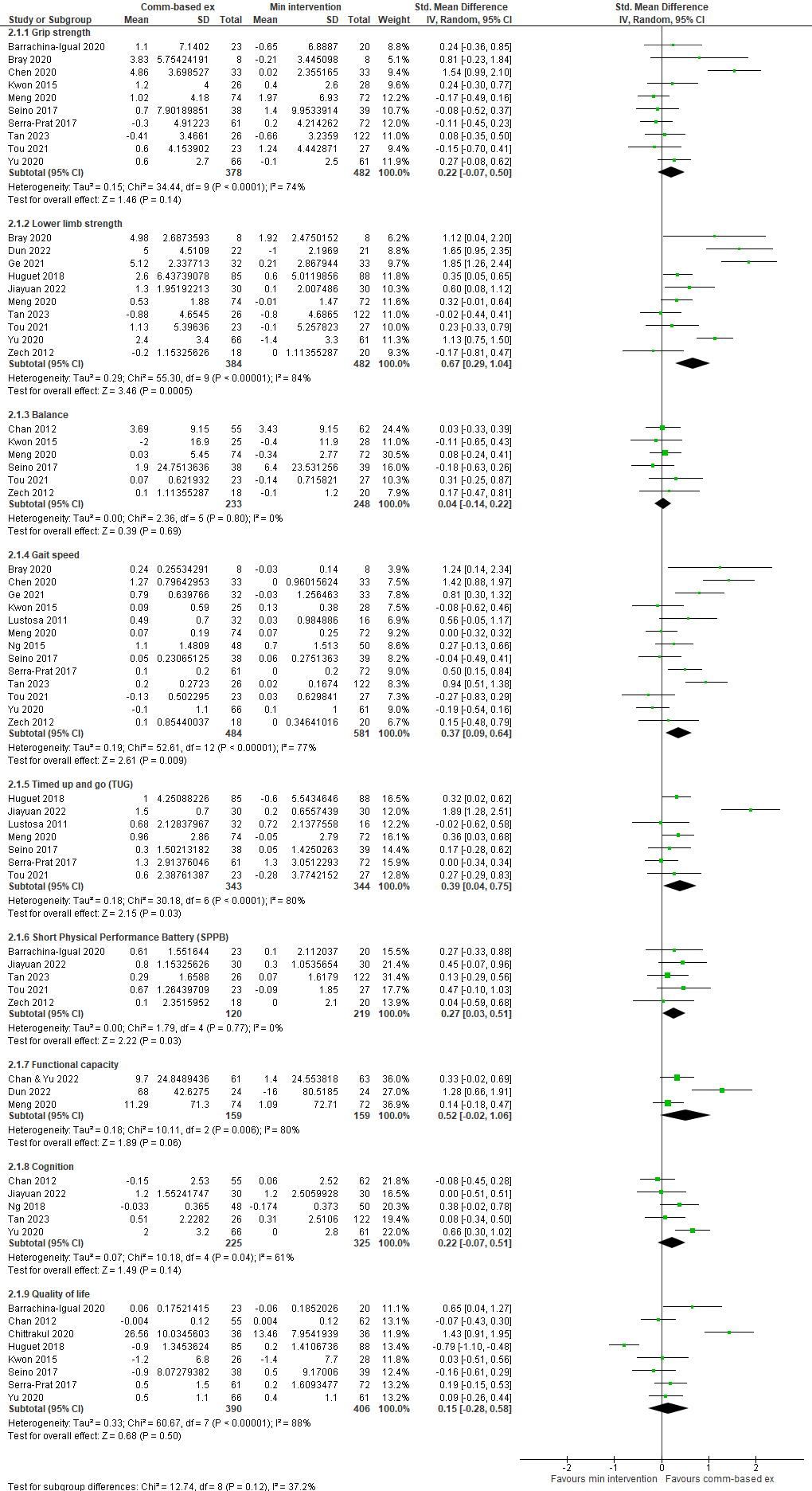


**Aii.** Pooled MD for measures with continuous data


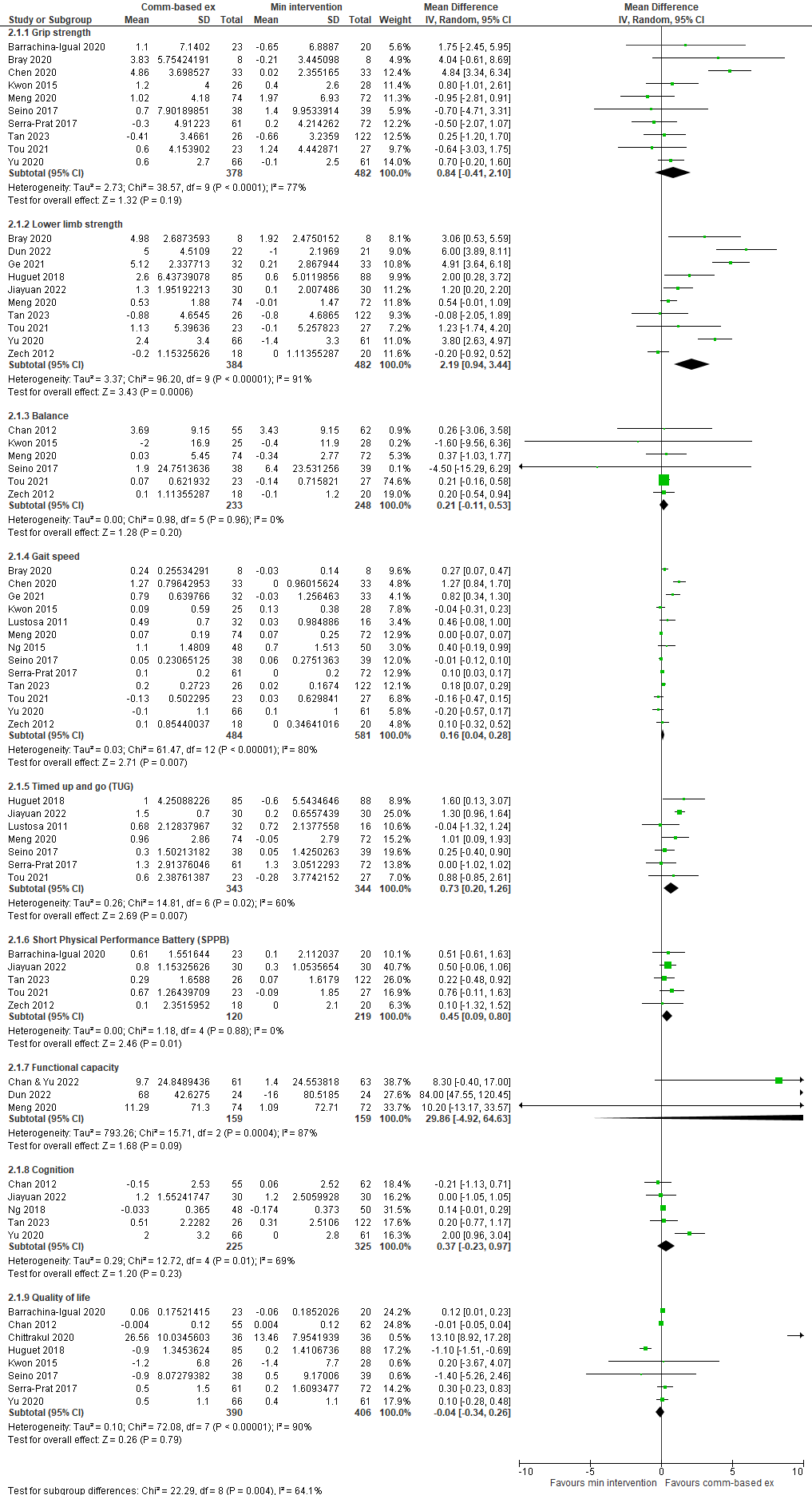


**B.** Pooled OR for measure with dichotomous data i.e. pre-frailty reversal


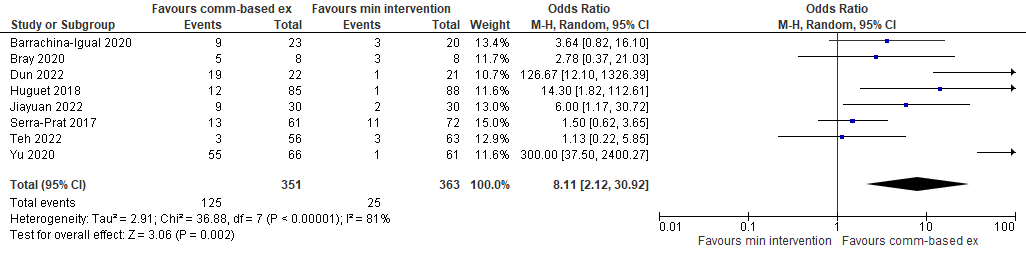

Supplement: Supplementary file 2 — Supplementary Material 2. [file 12877_2024_5150_MOESM2_ESM.docx]
