# Supplementary material for: Community-based exercises improve health status in pre-frail older adults: A systematic review with meta-analysis
Source: BMC Geriatr. 2024 Jul 10;24:589. doi: 10.1186/s12877-024-05150-7 (PMC11234756; doi:10.1186/s12877-024-05150-7)

**Supplementary 3:** Sub-group analyses based on exercise protocols.

**A.** Grip strength – Multi-component exercise only *versus* Minimal intervention


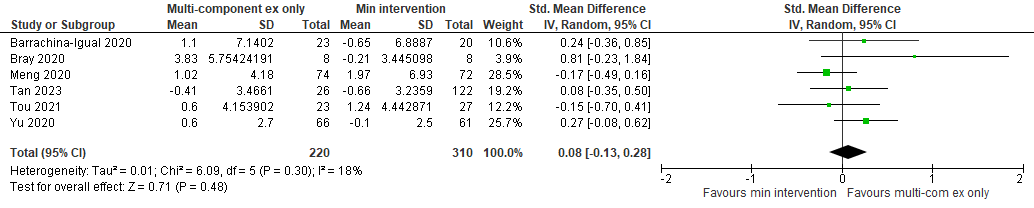


**B.** Lower limb strength – Multi-component exercise only *versus* Minimal intervention


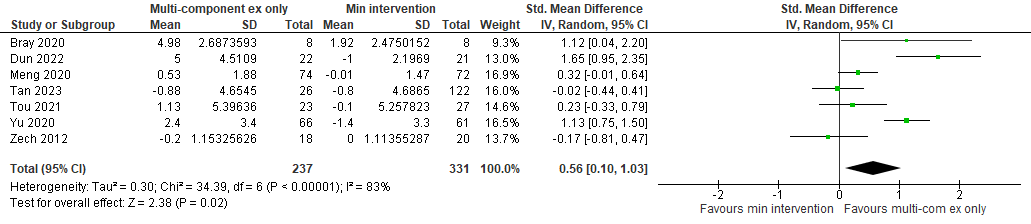


**C.** Balance – Multi-component exercise only *versus* Minimal intervention


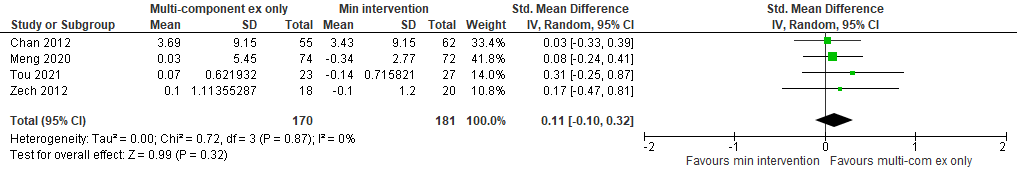


**D.** Gait speed – Multi-component exercise only *versus* Minimal intervention


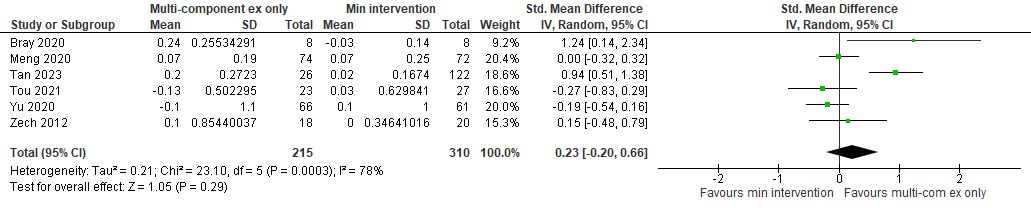


**E.** SPPB – Multi-component exercise only *versus* Minimal intervention


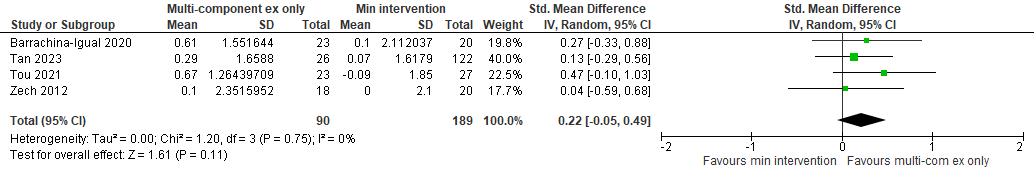


**F.** Cognition – Multi-component exercise only *versus* Minimal intervention


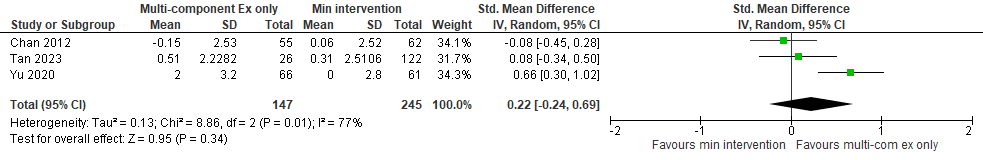


**Gi.** Quality of life – Multi-component exercise only *versus* Minimal intervention


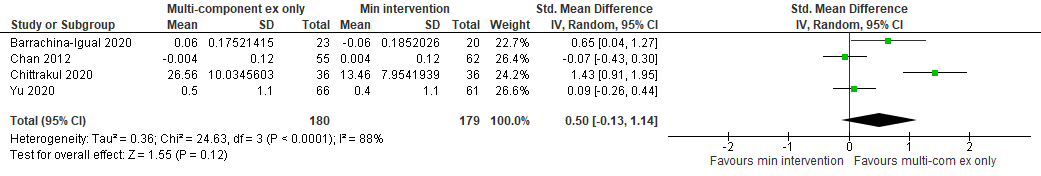


**Gii.** Quality of life – Multi-component exercise and nutrition *versus* Minimal intervention


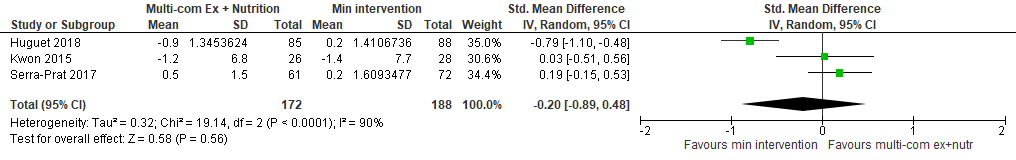


**Hi.** Pre-frailty reversal – Multi-component exercise only *versus* Minimal intervention


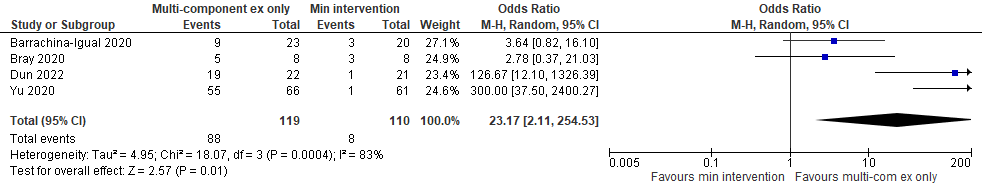


**Hii.** Pre-frailty reversal – Multi-component exercise and nutrition *versus* Minimal intervention


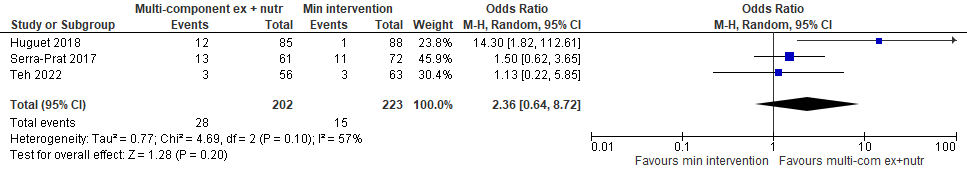

Supplement: Supplementary file 3 — Supplementary Material 3. [file 12877_2024_5150_MOESM3_ESM.docx]
