# Supplementary material for: Community-based exercises improve health status in pre-frail older adults: A systematic review with meta-analysis
Source: BMC Geriatr. 2024 Jul 10;24:589. doi: 10.1186/s12877-024-05150-7 (PMC11234756; doi:10.1186/s12877-024-05150-7)

**Supplementary 4:** Sub-group analyses based on lower limb strength measures

**Ai.** Pooled SMD for Timed 5-times Sit-to-stand


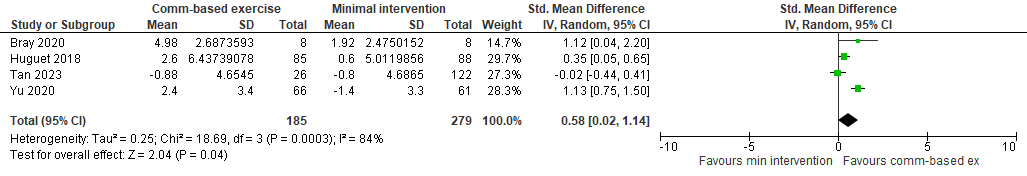


**Aii.** Pooled MD for Timed 5-times Sit-to-stand


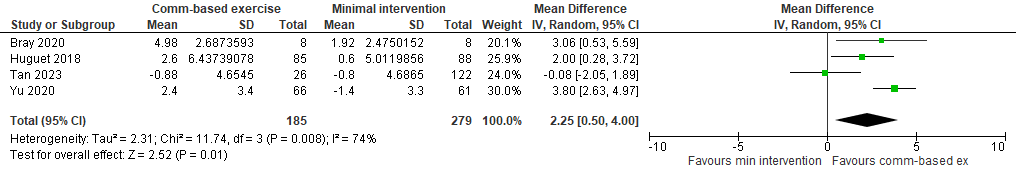


**Bi.** Pooled SMD for 30secs chair rise test


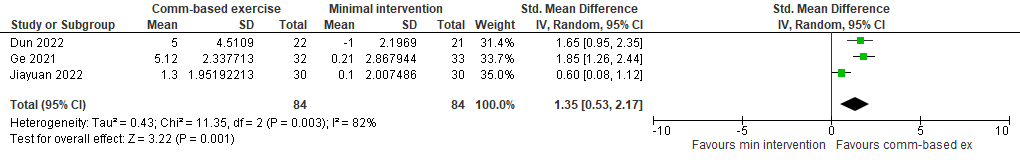


**Bii.** Pooled MD for 30secs chair rise test


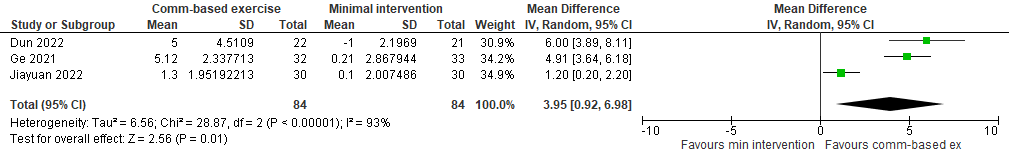

Supplement: Supplementary file 4 — Supplementary Material 4. [file 12877_2024_5150_MOESM4_ESM.docx]
