# Supplementary material for: Community-based exercises improve health status in pre-frail older adults: A systematic review with meta-analysis
Source: BMC Geriatr. 2024 Jul 10;24:589. doi: 10.1186/s12877-024-05150-7 (PMC11234756; doi:10.1186/s12877-024-05150-7)

**Supplementary 5:** Sub-group analyses based on cognitive measures

**Ai.** Pooled SMD (after excluding trials which used MMSE)


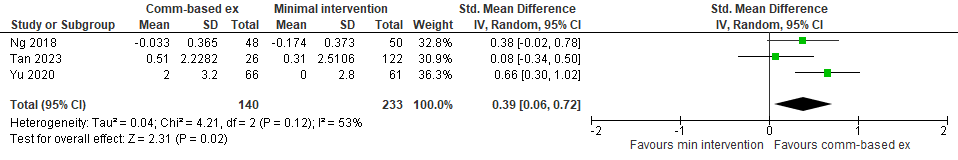


**Aii.** Pooled MD (after excluding trials which used MMSE)


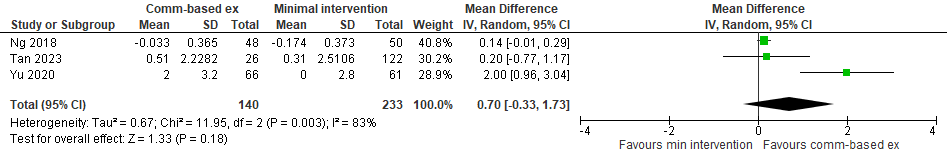

Supplement: Supplementary file 5 — Supplementary Material 5. [file 12877_2024_5150_MOESM5_ESM.docx]
