# Supplementary material for: Community-based exercises improve health status in pre-frail older adults: A systematic review with meta-analysis
Source: BMC Geriatr. 2024 Jul 10;24:589. doi: 10.1186/s12877-024-05150-7 (PMC11234756; doi:10.1186/s12877-024-05150-7)

**Supplementary 6:** Sub-group analyses based on quality of life measures

**Ai.** Pooled SMD for EQ-5D


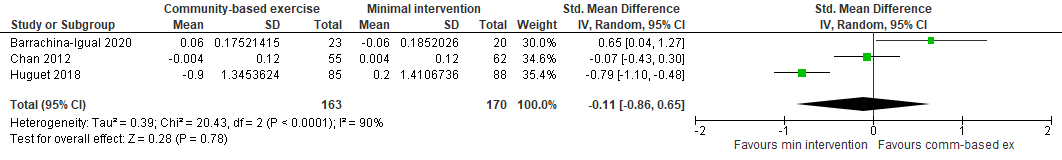


**Aii.** Pooled MD for EQ-5D


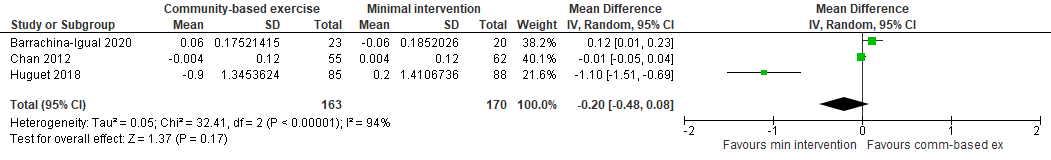


**Bi.** Pooled SMD for SF-36


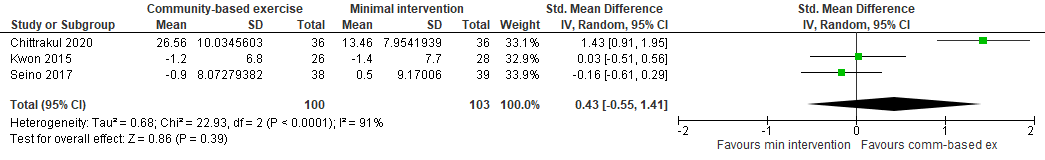


**Bii.** Pooled MD for SF-36


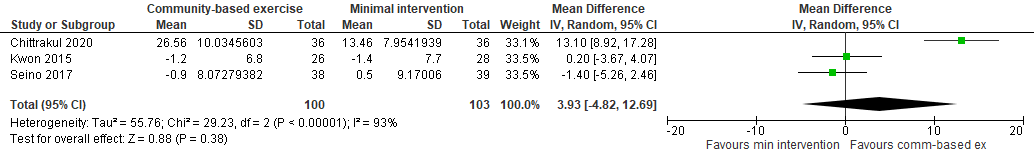

Supplement: Supplementary file 6 — Supplementary Material 6. [file 12877_2024_5150_MOESM6_ESM.docx]
